# Postsynaptic synucleins mediate endocannabinoid signaling

**DOI:** 10.1038/s41593-023-01345-0

**Published:** 2023-05-29

**Authors:** Eddy Albarran, Yue Sun, Yu Liu, Karthik Raju, Ao Dong, Yulong Li, Sui Wang, Thomas C. Südhof, Jun B. Ding

**Affiliations:** 1 Neurosciences Graduate Program, Stanford University, Stanford, CA USA; 2 Department of Neurosurgery, Stanford University, Stanford, CA USA; 3 Department of Molecular and Cellular Physiology, Stanford University and Howard Hughes Medical Institute, Stanford, CA USA; 4 State Key Laboratory of Membrane Biology, Peking University School of Life Sciences, Beijing, China; 5 PKU-IDG/McGovern Institute for Brain Research, Beijing, China; 6 Peking-Tsinghua Center for Life Sciences, Academy for Advanced Interdisciplinary Studies, Peking University, Beijing, China; 7 Chinese Institute for Brain Research, Beijing, China; 8 Department of Ophthalmology, Mary M. and Sash A. Spencer Center for Vision Research, Byers Eye Institute, Stanford University, Stanford, CA USA; 9 Department of Neurology and Neurological Sciences, Stanford University, Stanford, CA USA

**Keywords:** Long-term depression, Cellular neuroscience, Neurotransmitters

## Abstract

Endocannabinoids are among the most powerful modulators of synaptic transmission throughout the nervous system, and yet little is understood about the release of endocannabinoids from postsynaptic compartments. Here we report an unexpected finding that endocannabinoid release requires synucleins, key contributors to Parkinson’s disease. We show that endocannabinoids are released postsynaptically by a synuclein-dependent and SNARE-dependent mechanism. Specifically, we found that synuclein deletion blocks endocannabinoid-dependent synaptic plasticity; this block is reversed by postsynaptic expression of wild-type but not of mutant α-synuclein. Whole-cell recordings and direct optical monitoring of endocannabinoid signaling suggest that the synuclein deletion specifically blocks endocannabinoid release. Given the presynaptic role of synucleins in regulating vesicle lifecycle, we hypothesize that endocannabinoids are released via a membrane interaction mechanism. Consistent with this hypothesis, postsynaptic expression of tetanus toxin light chain, which cleaves synaptobrevin SNAREs, also blocks endocannabinoid-dependent signaling. The unexpected finding that endocannabinoids are released via a synuclein-dependent mechanism is consistent with a general function of synucleins in membrane trafficking and adds a piece to the longstanding puzzle of how neurons release endocannabinoids to induce synaptic plasticity.

## Abstract (web summary)

Endocannabinoids are important modulators of synaptic transmission, although their mechanism of release is unknown. In this study, the authors found that endocannabinoid postsynaptic release is mediated by synucleins via a synuclein-dependent and SNARE-dependent mechanism.

## Main

α-Synuclein (α-Syn) is a small protein that, together with the closely related β-Synuclein (β-Syn) and γ-Synuclein (γ-Syn), constitutes one of the most abundant proteins in the brain^1,2^. α-Syn plays a central role in Parkinson’s disease (PD) pathogenesis because α-Syn mutations and multiplications cause PD^3^; genome-wide association studies link α-Syn to sporadic forms of PD^4^; and the brains of patients with PD almost always contain Lewy bodies composed of α-Syn aggregate. However, the physiological function of α-Syn, and that of other synucleins, remains largely unknown.

Synucleins possess a conserved N-terminal domain that binds to phospholipids^5,6^, underlying α-Syn’s affinity for membranes such as synaptic vesicles^7,8^. Overexpression of α-Syn in vitro and in vivo inhibits exocytosis, possibly through impairments in synaptic vesicle endocytosis, recycling and dilation of the exocytotic fusion pore^8–11^. By contrast, deletion of α-Syn produces little to no effect on synaptic transmission, with α-Syn-knockout (KO) mice exhibiting only slight reductions in dopamine (DA) levels and displaying modest behavioral phenotypes^12^. Moreover, synuclein double-knockout (dKO) and triple-knockout (tKO) mice displayed no detectable changes in synaptic strength or short-term plasticity^13,14^. Thus, it has been difficult to reconcile α-Syn’s abundance and highly penetrant role in PD with its seemingly subtle endogenous function. Strikingly, even modest transgenic α-Syn overexpression completely prevents the lethality and neurodegeneration of CSPα KO mice^15^, suggesting an essential role for α-Syn in protection against neurodegeneration, which is counterintuitive given its causal involvement in PD.

The striatum, the input nucleus of the basal ganglia, is one of the most severely affected areas in PD, as the loss of DA signaling in the striatum and the degeneration of synapses on striatal spiny projection neurons (SPNs) greatly alter the striatal circuitry and underlie many of the motor and cognitive impairments observed in PD^16,17^. One particularly detrimental consequence of PD is the loss of endocannabinoid (eCB)-dependent plasticity at corticostriatal synapses^18,19^, which is central to striatum-dependent learning and habit formation^20–22^. In eCB-dependent plasticity, eCBs are synthesized and released postsynaptically in an activity-dependent and Ca^2+^-dependent manner. eCBs then retrogradely bind to presynaptic CB1 receptors (CB1Rs) to decrease the presynaptic release probability^23–26^. However, little is known about how eCBs are released from postsynaptic neurons. eCBs are amphiphilic molecules derived from phospholipids that are unlikely to diffuse passively out of the postsynaptic neurons and across the synaptic cleft^27^. Thus, it is unclear how eCBs reach presynaptic CB1Rs during synaptic plasticity, an essential step to understanding striatal function and eCB signaling.

## Results

### Basal synaptic transmission in Syn-tKO mice is unaffected

**Fig. 1:**
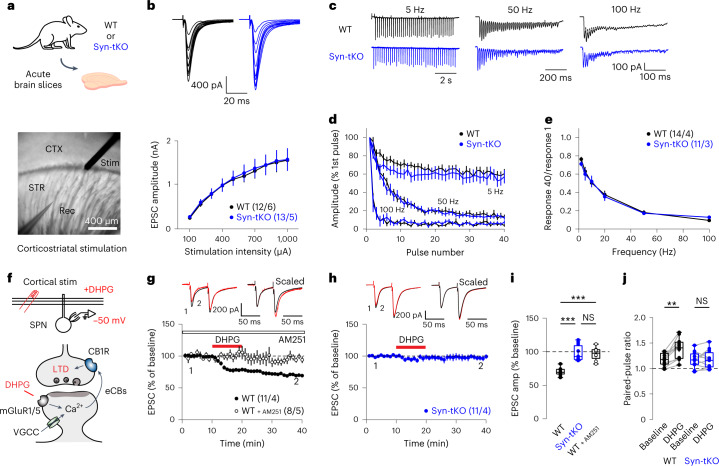
eCB-dependent LTD is abolished in Syn-tKO mice. **a**, Experimental configuration (bottom, one representative differential interference contrast (DIC) image of ~700 whole-cell recordings included in this study). **b**, Top, representative traces of evoked corticostriatal EPSCs across a range of stimulation intensities. Bottom, input–output curves in WT and Syn-tKO mice (WT: *n* = 12 cells, 6 mice; Syn-tKO: *n* = 13 cells, 6 mice; *P* = 0.888). **c**, Representative traces of responses to repeated stimulation across a range of stimulation frequencies. **d**,**e**, No difference in use-dependent synaptic properties in Syn-tKO mice, as measured by short-term depression dynamics (**d**; WT: *n* = 14 cells, 4 mice; Syn-tKO: *n* = 11 cells, 3 mice; 5 Hz: *P* = 0.304; 50 Hz: *P* = 0.651; 100 Hz: *P* = 0.691) and steady-state amplitudes (**e**; *P* = 0.756) in response to repeated stimulation across a range of frequencies. **f**, Schematic of eCB-LTD experiments. Top, whole-cell recordings from SPNs during induction of eCB-LTD; bottom, eCB-LTD induction with DHPG (50 µM) and depolarization (−50 mV). **g**–**j**, DHPG-induced eCB-LTD in WT mice is fully blocked by the CB1R antagonist AM251 (10 µM) (WT: *n* = 11 cells, 4 mice, 69.95 ± 1.70%; WT + AM251: *n* = 8 cells, 5 mice, 96.89 ± 3.42%; *P* = 6.375 × 10^−4^); top, representative traces. **h**, eCB-LTD is abolished in Syn-tKO mice (Syn-tKO: *n* = 11 cells, 4 mice, 97.76 ± 3.49%; *P* = 2.106 × 10^−4^); top, representative trace. **i**, Summary of EPSC amplitudes. **j**, Summary of PPRs (WT baseline: 1.18 ± 0.04; post-DHPG: 1.39 ± 0.06; *P* = 0.001; Syn-tKO baseline: 1.17 ± 0.05; post-DHPG: 1.19 ± 0.05; *P* = 0.831; WT + AM251 baseline: 1.26 ± 0.06; post-DHPG: 1.27 ± 0.06; *P* = 0.641). Data are mean ± s.e.m. **i**,**j**, Box plots are depicted as mean (center), first/third quartiles (lower/upper box limits) and minima/maxima (bottom/top whiskers). Statistical significance was assessed by two-sided tests, including two-way repeated-measures ANOVA with multiple comparisons (**b**,**d**,**e**), ANOVA with multiple comparisons (**i**) and Wilcoxon signed tests (**j**) (***P < 0.001; **P < 0.01; NS, not significant). Source data

**Extended Data Fig. 1:**
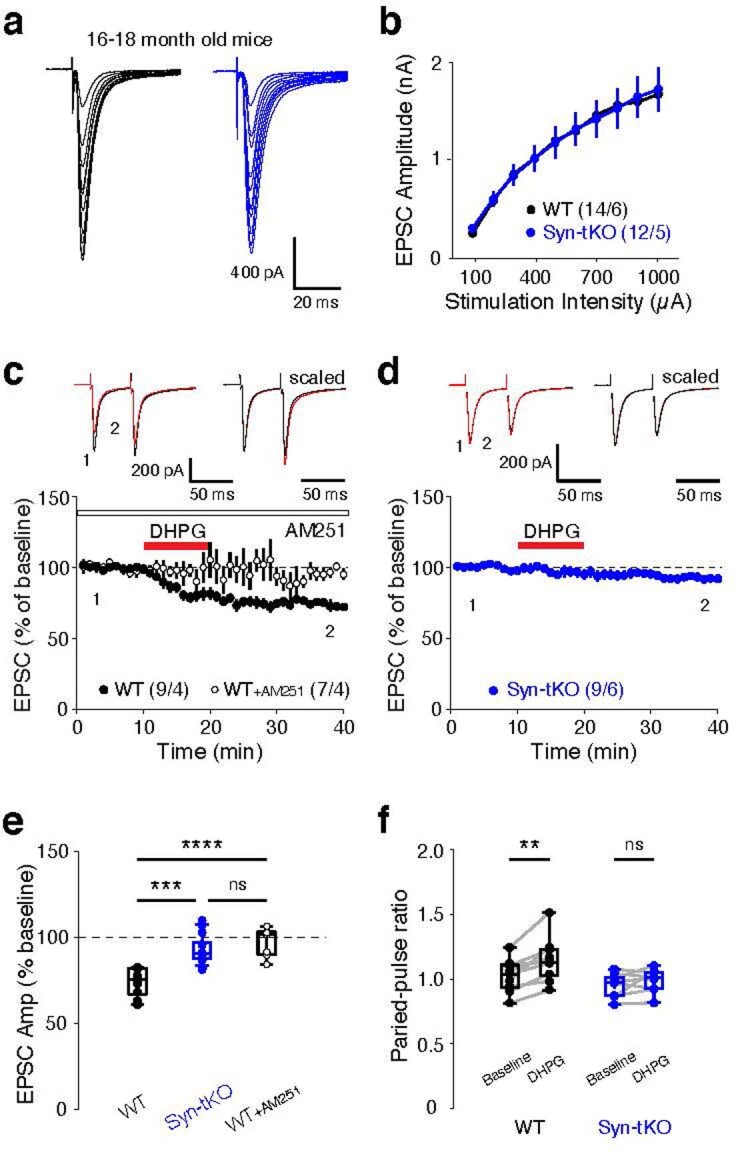
The triple α/β/γ-synuclein KO has no effect on basal corticostriatal synaptic transmission, but blocks eCB-LTD in aged Syn-tKO mice. **a**, Representative traces of evoked corticostriatal EPSCs in WT and Syn-tKO SPNs from aged mice (16–18 months old) across a range of stimulation intensities. **b**, Input-output curves in WT and Syn-tKO mice (WT: n = 14 cells / 6 mice; Syn-tKO: n = 12 cells / 5 mice; p = 0.960). **c-f**, DHPG-mediated eCB-LTD in aged (16–18 months old) WT mice is fully blocked by the CB1R antagonist AM251 (10 µM) (WT: n = 9 cells / 4 mice, 73.88 ± 2.74%; WT + AM251: n = 7 cells / 4 mice, 97.06 ± 3.20%; p = 3.026e-5); top, representative traces. **d**, eCB-LTD is abolished in aged Syn-tKO mice (Syn-tKO: n = 9 cells / 6 mice, 92.57 ± 2.57%; p = 1.955e-4); top, representative trace. **e**, Summary of EPSC amplitudes. **f**, Summary of PPRs (WT baseline: 1.02 ± 0.04; post-DHPG: 1.15 ± 0.06; p = 3.9e-3; Syn-tKO baseline: 0.95 ± 0.03; post-DHPG: 0.99 ± 0.03; p = 0.301). Data are mean ± SEM. (**e, f**) Box plots are depicted as mean (center), first/third quartile (lower/upper box limits), and minima/maxima (bottom/top whiskers). Statistical significance was assessed by two-sided tests, including 2-way repeated measures ANOVA (**b**), ANOVA with multiple comparisons (**e**), and Wilcoxon signed tests (**f**) (**** p < 0.0001; *** p < 0.001; ** p < 0.01; n.s. non-significant). Source data

Given the strong association of corticostriatal dysfunction with PD, we directly measured basal corticostriatal synaptic transmission and eCB-dependent plasticity in α/β/γ-synuclein tKO (Syn-tKO) mice. Previous reports suggested that α-Syn decreases neurotransmitter release by acting at presynaptic sites, with some studies showing increased synaptic transmission in single α-Syn KO mice^12^, whereas no such changes were detected in Syn-dKO^13^ or Syn-tKO mice^14^. We, therefore, investigated if corticostriatal synaptic transmission was abnormal in Syn-tKO mice. Whole-cell patch-clamp recordings from SPNs in acute slices of the dorsolateral striatum prepared from wild-type (WT) and Syn-tKO mice, combined with electrical stimulation of corticostriatal axons, allowed us to measure corticostriatal synaptic responses (Fig. 1a). We found no significant difference in the stimulus–response relationship between WT and Syn-tKO corticostriatal synapses (Fig. 1b). Because previous reports showed that survival and behavioral deficits are revealed at older ages in Syn-tKO mice^14,28^, we also tested aged mice (16–18 months old). Again, we observed no significant difference in synaptic strength between WT and Syn-tKO mice (Extended Data Fig. 1a,b).

We next measured the use-dependent dynamics of synaptic transmission by delivering stimulus trains at varying frequencies (Fig. 1c). We measured the rate of synaptic depression resulting from repeated stimulation^13^ and found virtually indistinguishable depression dynamics (Fig. 1d) and steady-state response amplitudes (Fig. 1e) between WT and Syn-tKO cells across stimulation frequencies. Together, these results show that basal corticostriatal synaptic transmission in Syn-tKO mice is largely normal, including responses engaged by repeated stimuli that depend on the rates of presynaptic vesicle recycling and the sizes of the reserve vesicle pool.

### Syn-tKO mice lack eCB-dependent plasticity

**Extended Data Fig. 2:**
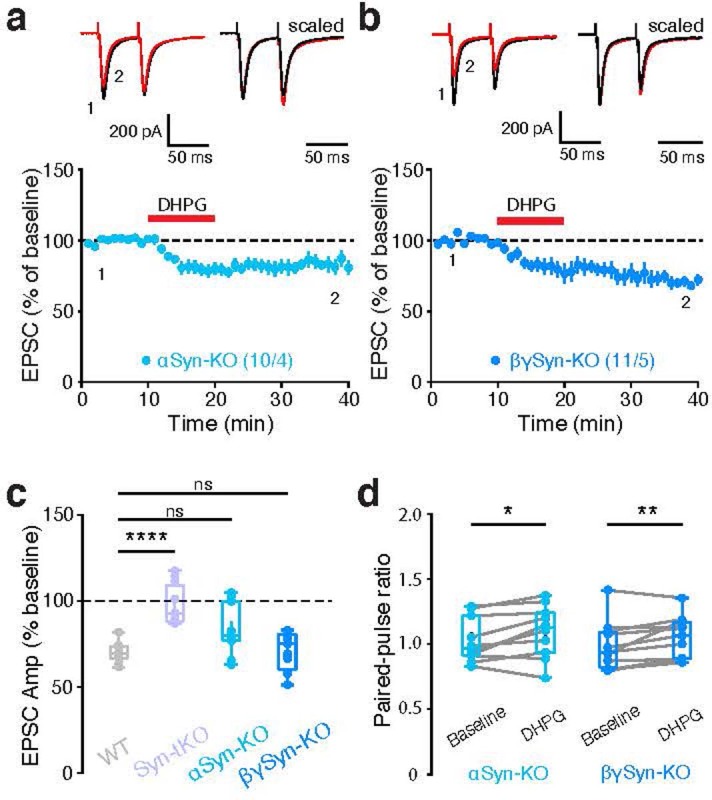
DHPG-LTD is not abolished in α-Syn-KO and βγ-Syn-KO mice. **a-d**, eCB-LTD in α-Syn-KO (WT data from Fig. 1: n = 11 cells / 4 mice, 69.95 ± 1.70%; α-Syn-KO: n = 10 cells / 4 mice, 83.44 ± 4.67%; p = 0.162). **b**, eCB-LTD in βγ-Syn-KO mice (n = 11 cells / 5 mice, 71.00 ± 3.38%; p = 0.957). **c**, Summary of EPSC amplitudes. **d**, Summary of PPRs in α-Syn-KO (baseline: 1.03 ± 0.05; post-DHPG: 1.10 ± 0.06; p = 0.037) and βγ-Syn-KO mice (baseline: 0.98 ± 0.06; post-DHPG: 1.05 ± 0.05; p = 9.8e-3). Data are mean ± SEM. (**c, d**) Box plots are depicted as mean (center), first/third quartile (lower/upper box limits), and minima/maxima (bottom/top whiskers). Statistical significance was assessed by two-sided tests, including ANOVA with multiple comparisons (**c**), and Wilcoxon signed tests (**d**) (**** p < 0.0001; ** p < 0.01; * p < 0.05; n.s. non-significant). Source data

One of the best-characterized forms of corticostriatal synaptic plasticity is eCB-long-term depression (LTD)^29–31^, which is required for striatal learning^20,22^. Importantly, impairments in corticostriatal eCB-LTD are observed in mouse models of PD^19,32^. We assayed eCB-LTD in acute slices of young adult (3 months old) WT and Syn-tKO mice by combining slight membrane depolarization (−50 mV) with an application of a type I mGluR agonist ((S)-3,5-dihydroxyphenylglycine (DHPG, 50 µM); Fig. 1f), which results in a lasting depression of evoked corticostriatal excitatory postsynaptic currents (EPSCs) (Fig. 1g). Strikingly, we found that eCB-LTD is abolished in Syn-tKO mice (Fig. 1h). Syn-tKO cells were indistinguishable from WT cells in the presence of the CB1R antagonist AM251 (10 µM) (Fig. 1g,i). Notably, paired-pulse ratios (PPRs) were significantly increased in WT cells after eCB-LTD but not in Syn-tKO cells (Fig. 1j), consistent with a selective decrease in presynaptic release probability in WT cells. We observed impaired eCB-LTD in both young adult and aged mice (16–18 months old; Extended Data Fig. 1c–f), suggesting that the phenotype is not an age-dependent effect but, instead, due to a direct loss of an endogenous synuclein function. Furthermore, we found that eCB-LTD was normally expressed in KO mice lacking α-Syn alone or both β-synuclein and γ-synuclein (βγ-Syn-KO mice), suggesting redundancy among synucleins (Extended Data Fig. 2a–d).

**Fig. 2:**
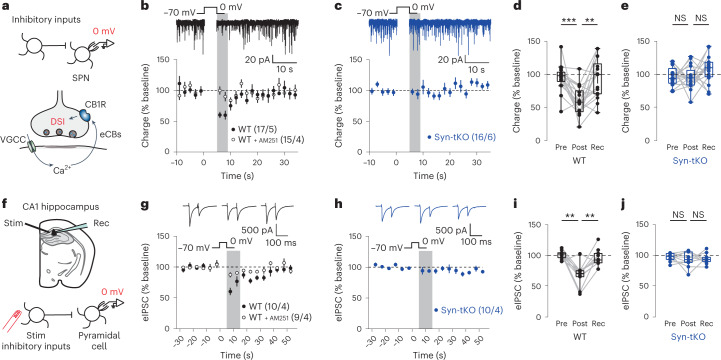
DSI is abolished in Syn-tKO mice. **a**, Schematic of DSI experiments. Top, whole-cell recordings from SPNs during induction of DSI; bottom, DSI induction pathway engaged with depolarization (depol) (0 mV). **b**, DSI in WT mice is blocked by AM251 (10 µM); top, representative WT recorded trace. **c**, DSI is abolished in Syn-tKO mice; top, representative Syn-tKO recorded trace. **d**, Summary of DSI for WT mice (*n* = 17 cells, 5 mice, pre-depol: 95.64 ± 5.01%, post-depol: 59.91 ± 5.28%, recovery: 93.51 ± 7.20%, *P* = 5 × 10^−4^, *P* = 1.6 × 10^−3^). **e**, Summary of DSI for Syn-tKO mice (*n* = 16 cells, 6 mice, pre-depol: 97.25 ± 3.66%, post-depol: 95.70 ± 4.54%, recovery: 107.59 ± 5.20%, *P* = 0.959, *P* = 0.148). **f**, Schematic of DSI experiments in CA1 of the hippocampal pyramidal neurons. **g**,**i**, Summary of DSI in WT mice (*n* = 10 cells, 4 mice; pre-depol: 101.50 ± 2.18%; post-depol: 68.26 ± 5.85%; recovery: 96.77 ± 4.36%; *P* = 3.9 × 10^−3^, *P* = 3.9 × 10^−3^). **h**,**j**, Summary of DSI in Syn-tKO (*n* = 10 cells, 4 mice; pre-depol: 97.24 ± 2.08%; post-depol: 93.70 ± 3.94%; recovery: 94.73 ± 2.72%; *P* = 0.625, *P* = 0.846). Data are mean ± s.e.m. **d**,**e**,**i**,**j**, Box plots are depicted as mean (center), first/third quartiles (lower/upper box limits) and minima/maxima (bottom/top whiskers). Statistical significance was assessed by two-sided tests, including Wilcoxon signed tests (**d**,**e**,**i**,**j**) (***P < 0.001; **P < 0.01; NS, not significant). Rec, recovery. Source data

**Extended Data Fig. 3:**
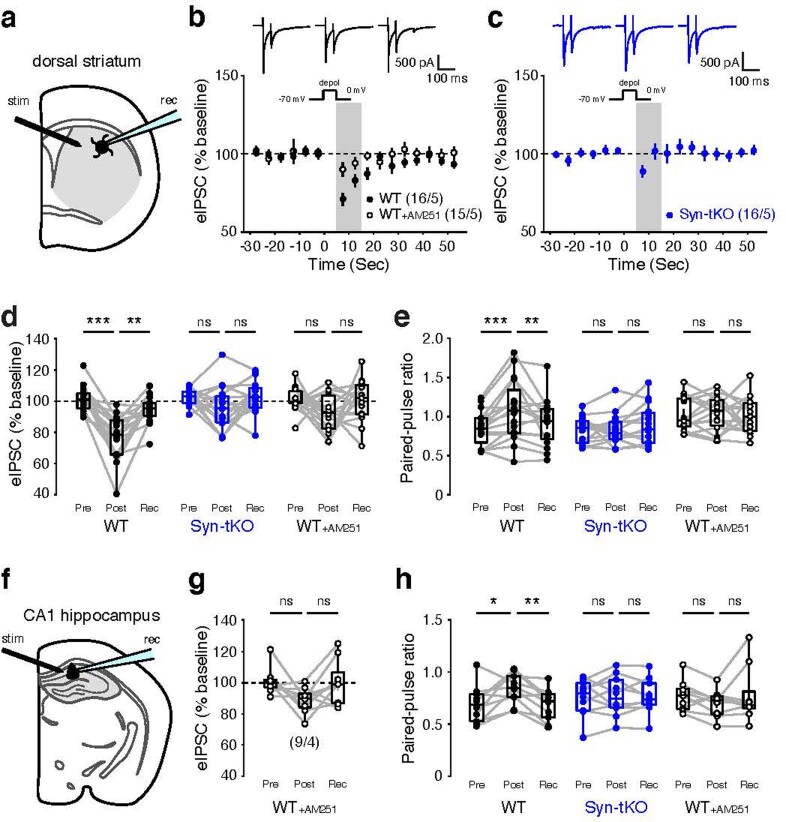
DSI is abolished in dorsal striatum SPNs and hippocampal CA1 pyramidal neurons of Syn-tKO mice. **a**, Schematic of evoked DSI experiments in the dorsal striatum. **b-e**, DSI (evoked-IPSCs) in dorsal striatum SPNs of WT mice (n = 16/5; pre-depol: 100.94 ± 2.09%; post-depol: 76.95 ± 3.64%; recovery: 94.43 ± 2.16%; p = 4.378e-4, p = 7.764e-4). DSI is blocked by AM251 (10 µM) (n = 15/5; pre-depol: 101.15 ± 1.99%; post-depol: 92.13 ± 3.14%; recovery: 99.97 ± 3.50%; p = 0.055, p = 0.277). **c**, Striatal DSI in Syn-tKO mice (n = 16/5; pre-depol: 102.19 ± 1.29%; post-depol: 95.25 ± 3.54%; recovery: 101.64 ± 2.53%; p = 0.134, p = 0.134). **d**, Summary of IPSC amplitudes. **e**, Summary of PPRs during striatal DSI (WT pre-depol: 0.85 ± 0.06; post-depol: 1.08 ± 0.10; recovery: 0.92 ± 0.07; p = 2.3e-3, p = 0.030; WT + AM251 pre-depol: 1.04 ± 0.06; post-depol: 1.04 ± 0.06; recovery: 1.01 ± 0.06; p = 0.847, p = 0.600; Syn-tKO pre-depol: 0.83 ± 0.04; post-depol: 0.82 ± 0.05; recovery: 0.89 ± 0.06; p = 0.959, p = 0.134). **f**, Schematic of DSI experiments in CA1 neurons. **g**, Summary of DSI with AM251 (n = 9/4; pre-depol: 101.17 ± 2.82%; post-depol: 89.22 ± 2.67%; recovery: 100.26 ± 4.74%; p = 0.055, p = 0.098). **h**, Summary of PPRs during hippocampal DSI (WT n = 10/4; pre-depol: 0.69 ± 0.06; post-depol: 0.84 ± 0.05; recovery: 0.69 ± 0.05; p = 0.037, p = 5.9e-3; WT + AM251 n = 9/4; pre-depol: 0.77 ± 0.05; post-depol: 0.70 ± 0.04; recovery: 0.78 ± 0.09; p = 0.098, p = 0.570; Syn-tKO n = 10/4; pre-depol: 0.75 ± 0.06; post-depol: 0.77 ± 0.06; recovery: 0.77 ± 0.05; p = 0.770, p = 1.000). Data are mean ± SEM. n = cells / mice. (**d, e, g, h**) Box plots are depicted as mean, first/third quartile, and minima/maxima. Statistical significance was assessed by two-sided Wilcoxon signed tests (**d, e, g, h**) (*** p < 0.001; ** p < 0.01; * p < 0.05; n.s. non-significant). Source data

To further characterize the Syn-tKO phenotype, we measured depolarization-induced suppression of inhibition (DSI)^26^, a different form of eCB-dependent plasticity in the striatum. During DSI, strong depolarization of SPNs results in the Ca^2+^-dependent synthesis and release of eCBs that transiently suppress inhibitory inputs (Fig. 2a)^23–25^. Indeed, a 5-s depolarization (to 0 mV) in WT cells was sufficient to transiently inhibit spontaneous inhibitory postsynaptic currents (sIPSCs) in a CB1R-dependent manner (Fig. 2b,d). Strikingly, the same DSI protocol failed to elicit a significant reduction in sIPSCs in Syn-tKO mice (Fig. 2c–e). We observed the same results when we repeated this experiment using a stimulation-evoked IPSC protocol (Extended Data Fig. 3a–d), with WT but not Syn-tKO cells showing a significant increase in PPRs during DSI (Extended Data Fig. 3e), which reflects the presynaptic locus of the transient suppression of inhibitory inputs.

Finally, in a parallel set of experiments, we recorded DSI in pyramidal neurons of the hippocampal CA1 region (Fig. 2f)^25^. Here, we once again found that DSI was readily inducible in WT cells but not in Syn-tKO cells (Fig. 2g–j and Extended Data Fig. 3f–h). The observations that Syn-tKO mice exhibit impairments in two forms of eCB plasticity (eCB-LTD and DSI), across different synapse types (glutamatergic and GABAergic) and brain regions (striatum and hippocampus), suggest a broad defect in eCB signaling in Syn-tKO mice.

### Presynaptic CB1Rs are intact in Syn-tKO mice

**Fig. 3:**
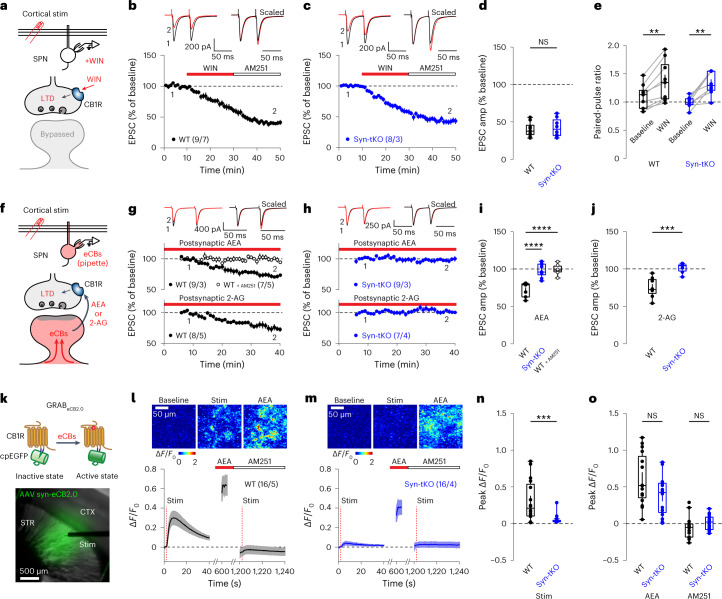
eCB release is impaired in Syn-tKO mice. **a**, Schematic of WIN-LTD experiments. **b**–**d**, Normal WIN-LTD in WT and Syn-tKO mice (WT: *n* = 9/7, 39.91 ± 3.18%; Syn-tKO: *n* = 8/3, 42.46 ± 4.36%; *P* = 0.673). **e**, PPRs in WT mice (baseline: 1.09 ± 0.07; post-WIN: 1.36 ± 0.12; *P* = 3.9 × 10^−3^) and Syn-tKO mice (baseline: 0.99 ± 0.04; post-WIN: 1.33 ± 0.07; *P* = 7.8 × 10^−3^). **f**, Schematic of eCB-loading experiments. **g**–**j**, eCB-loading causes LTD in WT cells, blocked by AM251 (AEA WT: *n* = 9/3, 72.40 ± 3.10%; AEA WT + AM251: *n* = 7/5, 99.13 ± 2.63%; *P* = 1.08 × 10^−5^; 2-AG WT: *n* = 8/5, 74.92 ± 4.66%). **h**, eCB loading fails to induce LTD in Syn-tKO cells (AEA Syn-tKO: *n* = 9/3, 98.16 ± 3.05; *P* = 7.08 × 10^−6^; 2-AG Syn-tKO: *n* = 7/4, 101.22 ± 2.46%; *P* = 6.21 × 10^−4^). eCB loading increases PPRs in WT cells (AEA baseline: 1.02 ± 0.04; end: 1.19 ± 0.05; *P* = 3.9 × 10^−3^; 2-AG baseline: 1.26 ± 0.08; end: 1.38 ± 0.09; *P* = 7.8 × 10^−3^), not Syn-tKO cells (AEA baseline: 1.00 ± 0.04; end: 1.00 ± 0.05; *P* = 0.82; 2-AG baseline: 1.44 ± 0.07; end: 1.45 ± 0.06; *P* = 0.81). **k**, Top, GRAB_eCB2.0_ activation; bottom, representative image (1/32) of a GRAB_eCB2.0_-expressing slice. **l**, Stimulation increases GRAB_eCB2.0_ signal in WT condition; top, representative images. **m**–**o**, Stimulation-evoked GRAB_eCB2.0_ transients in Syn-tKO slices are reduced (WT: *n* = 16/5, 0.31 ± 0.07 Δ*F*/*F*_0_; Syn-tKO: *n* = 16/4, 0.05 ± 0.02 Δ*F*/*F*_0_; *P* = 8.52 × 10^−4^). **l**,**m**,**o**, No differences between WT and Syn-tKO slices with AEA (WT: 0.61 ± 0.09 Δ*F*/*F*_0_; Syn-tKO: 0.37 ± 0.07 Δ*F*/*F*_0_; *P* = 0.073) or AM251 (WT: −0.05 ± 0.04 Δ*F*/*F*_0_; Syn-tKO: 0.02 ± 0.03 Δ*F*/*F*_0_; *P* = 0.086). Data are mean ± s.e.m. *n* = cells or slices per mouse. **d**,**e**,**i**,**j**,**n**,**o**, Box plots are depicted as mean, first/third quartiles and minima/maxima. Significance was assessed by two-sided tests: Mann–Whitney tests (**d**,**j**,**n**,**o**), Wilcoxon signed tests (**e**) and ANOVA with multiple comparisons (**i**) (****P < 0.0001; ***P < 0.001; **P < 0.01; NS, not significant). Source data

**Extended Data Fig. 4:**
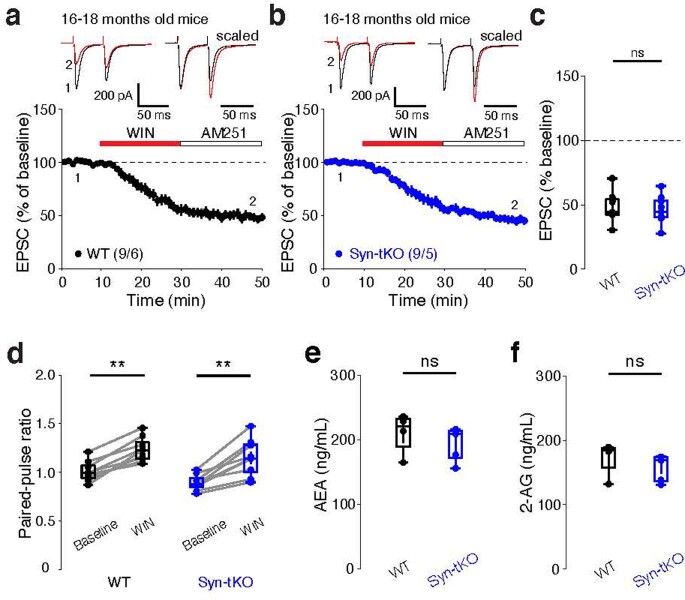
Syn-tKO has no effect on presynaptic WIN-LTD in aged mice and does not change the total AEA or 2-AG levels in the brain. **a-d**, WIN application results in indistinguishable corticostriatal LTD in aged (16–18 months) WT (**a**) and aged Syn-tKO mice (**b**) (WT: n = 9 cells / 6 mice, 48.11 ± 3.78%; Syn-tKO: n = 9 cells / 5 mice, 46.01 ± 3.56%; p = 0.605). **c**, Summary of EPSC amplitudes. **d**, Summary of PPRs in aged WT (baseline: 1.02 ± 0.03; post-WIN: 1.24 ± 0.04; p = 3.9e-3) and aged Syn-tKO mice (baseline: 0.90 ± 0.03; post-WIN: 1.16 ± 0.06; p = 3.9e-3). **e**, AEA levels detected in brain tissue of WT and Syn-tKO mice (WT: n = 4 mice, 211.08 ± 16.17 ng/mL; Syn-tKO: n = 5 mice, 194.40 ± 11.94 ng/mL; p = 0.286). **f**, 2-AG levels detected in brain tissue of WT and Syn-tKO (WT: 172.57 ± 13.55 ng/mL; Syn-tKO: 157.38 ± 9.34 ng/mL; p = 0.191). Data are mean ± SEM. (**c, d, e, f**) Box plots are depicted as mean (center), first/third quartile (lower/upper box limits), and minima/maxima (bottom/top whiskers). Statistical significance was assessed by two-sided tests, including Mann-Whitney (**c, e, f**) and Wilcoxon signed tests (**d**) (** p < 0.01; n.s. non-significant). Source data

α-Syn is thought to function predominantly in the presynaptic terminal, suggesting that the impairment in eCB-dependent synaptic plasticity in Syn-tKO mice is likely due to a failure of CB1R signaling^33^. To test this hypothesis, we applied the CB1R agonist WIN55,212 (WIN, 2 µM) in acute brain slices. WIN strongly depressed corticostriatal transmission via direct activation of presynaptic CB1Rs, bypassing the postsynaptic eCB synthesis and release mechanisms engaged during eCB-LTD and DSI (Fig. 3a). We found that WIN strongly reduced evoked EPSCs in both WT and Syn-tKO mice (Fig. 3b,c). The magnitude of synaptic depression was indistinguishable between genotypes (Fig. 3d) as was the concomitant significant increase in PPRs (Fig. 3e) that would be expected for a presynaptic weakening via CB1R activation. These results were reproduced when repeated in aged mice (Extended Data Fig. 4a–d). Thus, presynaptic CB1R function is intact in Syn-tKO mice, suggesting a postsynaptic deficit upstream of CB1R activation.

### Release of eCBs is impaired in Syn-tKO mice

Given the defects in eCB plasticity across different experimental contexts, we next tested whether a more upstream step in eCB signaling was impaired in Syn-tKO mice. We measured baseline levels of the eCBs anandamide (AEA) and 2-arachidonoylglycerol (2-AG) in striatal tissue and found no difference between WT and Syn-tKO (Extended Data Fig. 4e,f). We, therefore, focused on the postsynaptic release of eCBs as retrograde signals, which precedes CB1R activation but is downstream of eCB synthesis^34^. Although the specific mechanisms of retrograde eCB release are not well understood, the direct introduction of eCBs into a postsynaptic neuron via a patch pipette has been shown to induce a progressive release of these eCBs, resulting in synaptic depression^35^. Thus, to directly test eCB release, we dialyzed SPNs intracellularly with AEA (50 µM) or 2-AG (50 µM) through the patch pipette (Fig. 3f). In WT cells, the intracellular application of AEA or 2-AG caused a progressive depression of evoked corticostriatal EPSCs that depended on CB1R function (Fig. 3g). Strikingly, in Syn-tKO cells, postsynaptic AEA or 2-AG loading had no effect (Fig. 3h–j). Because intracellular loading with eCBs bypasses the eCB synthesis pathways, these results suggest that the defect in Syn-tKO mice lies specifically in the release of eCBs from postsynaptic cells.

To directly visualize eCB release, we used a recently developed eCB fluorescent sensor (GRAB_eCB2.0_)^36^. Viral expression of the GRAB_eCB2.0_ sensor in the dorsal striatum of mice allowed us to image stimulation-induced release of eCBs in acute slices (Fig. 3k). Local electrical stimulation in WT slices resulted in a significant increase in GRAB_eCB2.0_ signal, reflecting the release of eCBs (Fig. 3l). However, evoked GRAB_eCB2.0_ signals were significantly reduced in Syn-tKO mice (Fig. 3m,n), consistent with a deficit in eCB release. Notably, we validated GRAB_eCB2.0_ sensor expression and function in all imaged slices. Bath application of AEA (10 µM) significantly increased GRAB_eCB2.0_ fluorescence in both WT and Syn-tKO mice, and AM251 (10 µM) decreased GRAB_eCB2.0_ fluorescence and blocked stimulation-induced GRAB_eCB2.0_ activity in WT slices (Fig. 3l–o). Thus, in combination with our electrophysiology data, these results suggest that normal eCB release requires synucleins.

### eCB plasticity requires postsynaptic α-Syn

**Fig. 4:**
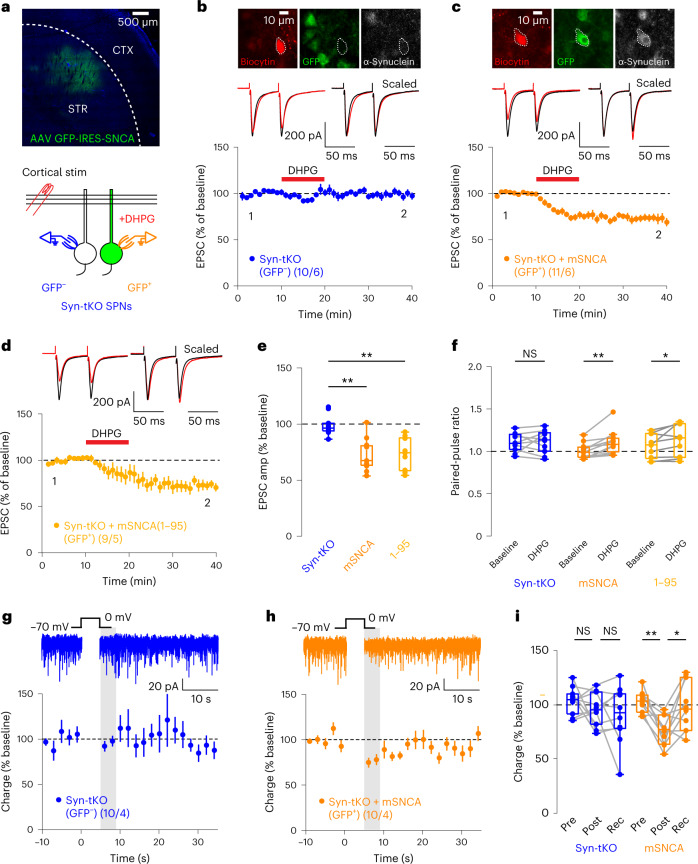
Postsynaptic α-Syn rescues eCB plasticity in Syn-tKO mice by a cell-autonomous mechanism. **a**, Experimental approach. Top, AAV-mediated expression of α-Syn and GFP in dorsolateral striatum of Syn-tKO mice; bottom, Syn-tKO (GFP^−^) and α-Syn-expressing SPNs (GFP^+^) targeted for recordings. **b**,**c**, Top, Post hoc verification of α-Syn expression. **b**–**f**, Postsynaptic expression of full-length α-Syn in Syn-tKO cells is sufficient to rescue eCB-LTD (GFP^–^, pooled: *n* = 10 cells, 6 mice, 99. 04 ± 2.89%; GFP^+^, mSNCA: *n* = 11 cells, 6 mice, 72.71 ± 4.32%; *P* = 2.09 × 10^−4^). **d**, Postsynaptic expression of a C-terminus-truncated α-Syn (1–95) still rescued eCB-LTD in Syn-tKO mice (GFP^+^, 1–95: *n* = 9 cells, 5 mice, 73.47 ± 4.84%; *P* = 5.41 × 10^−4^). **e**, Summary of EPSC amplitudes. **f**, Summary of PPRs in full-length α-Syn-expressing cells (GFP^+^, mSNCA; baseline: 1.01 ± 0.03; post-DHPG: 1.11 ± 0.04; *P* = 2.0 × 10^−3^), C-terminus-truncated α-Syn-expressing cells (GFP^+^, 1–95; baseline: 1.07 ± 0.05; post-DHPG: 1.14 ± 0.06; *P* = 0.039) and GFP^−^ cells (GFP^−^, pooled; baseline: 1.08 ± 0.03; post-DHPG: 1.09 ± 0.04; *P* = 1.0). **g**–**i**, Postsynaptic expression of full-length α-Syn rescues DSI in Syn-tKO SPNs (GFP^−^ cells: *n* = 10 cells, 4 mice, pre-depol: 103.58 ± 4.01%, post-depol: 95.81 ± 4.88%, recovery: 90.66 ± 8.44%, *P* = 0.695, *P* = 0.770; GFP^+^ cells: *n* = 10 cells, 4 mice, pre-depol: 102.73 ± 3.19%, post-depol: 76.21 ± 4.29%, recovery: 98.32 ± 7.27%, *P* = 3.9 × 10^−3^, *P* = 0.027). **i**, Summary of DSI. Data are mean ± s.e.m. **e**,**f**,**i**, Box plots are depicted as mean (center), first/third quartiles (lower/upper box limits) and minima/maxima (bottom/top whiskers). Statistical significance was assessed by two-sided tests, including Wilcoxon signed tests (**f**,**i**) and ANOVA with multiple comparisons (**e**) (**P < 0.01; *P < 0.05; NS, not significant). depol, depolarization. Rec, recovery. Source data

Thus far, our results suggest that synucleins are required for the postsynaptic release of eCBs. To further test this conclusion, we sparsely infected SPNs in the dorsolateral striatum of Syn-tKO mice with adeno-associated viruses (AAVs) that co-express green fluorescent protein (GFP) and α-Syn (Fig. 4a, top). Recordings of corticostriatal eCB-LTD from GFP^+^ or GFP^−^ cells allowed us to directly test whether postsynaptic exogenous α-Syn can rescue the Syn-tKO phenotype (Fig. 4a, bottom). As expected, eCB-LTD was not observed in GFP^−^ cells (Fig. 4b). Remarkably, almost all GFP^+^ cells expressing α-Syn exhibited significant eCB-LTD (10 of 11) (Fig. 4c,e). The presence or absence of α-Syn in recorded cells was confirmed by immunocytochemistry (Fig. 4b,c, top). Moreover, viral expression of C-terminally truncated α-Syn (residues 1–95) also rescued eCB-LTD in Syn-tKO cells (Fig. 4d,e). The rescued eCB-LTD in GFP^+^ cells was accompanied by a significant increase in PPRs, which was not observed in uninfected GFP^−^ cells (Fig. 4f). Finally, postsynaptic rescue of α-Syn also restored striatal DSI in Syn-tKO cells (Fig. 4g–i). Together, these results demonstrate that, not only are synucleins required for eCB plasticity, but also that the role they play is a postsynaptic one.

### α-Syn membrane-binding domains are needed for eCB plasticity

**Extended Data Fig. 5:**
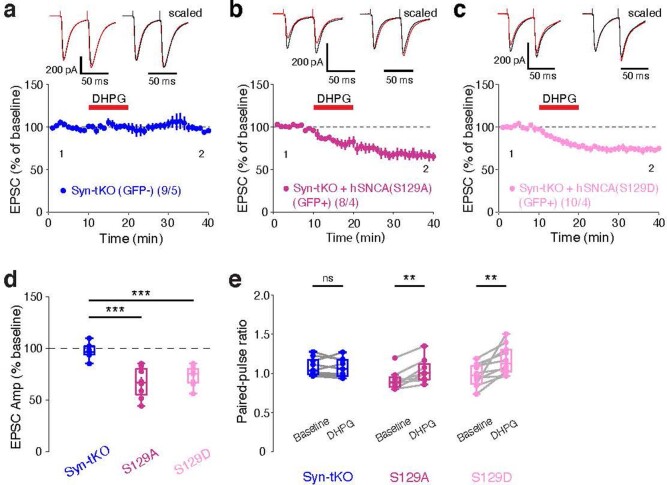
α-Syn S129 phosphorylation site mutations do not disrupt rescue of DHPG-induced eCB-LTD. **a**, eCB-LTD recorded in GFP- SPNs in Syn-tKO mice**. b, c**, eCB-LTD recorded from SPNs expressing α-Syn S129A (**b**) nor S129D (**c**) mutations. **d,e**, Summary of EPSC amplitudes. Postsynaptic expression of either S129 mutant α-Syn can rescue eCB-LTD (GFP-, pooled: n = 9 cells / 5 mice, 97.54 ± 2.28%; GFP+, S129A: n = 8 cells / 4 mice, 66.89 ± 5.24%; p = 1.03e-5; GFP+, S129D: n = 10 cells / 4 mice, 73.62 ± 2.89%, p = 1.39e-4). **e**, Summary of PPRs (S129A baseline: 0.91 ± 0.05; post-DHPG: 1.04 ± 0.06; p = 7.8e-3; S129D baseline: 0.98 ± 0.04; post-DHPG: 1.18 ± 0.06; p = 2.0e-3; GFP- (pooled) baseline: 1.08 ± 0.04; post-DHPG: 1.07 ± 0.04; p = 0.496). Data are mean ± SEM. (**d, e**) Box plots are depicted as mean (center), first/third quartile (lower/upper box limits), and minima/maxima (bottom/top whiskers). Statistical significance was assessed by two-sided tests, including ANOVA with multiple comparisons (**d**) and Wilcoxon signed tests (**e**) (*** p < 0.001; ** p < 0.01; n.s. non-significant). Source data

To dissect the mechanism of synuclein function in postsynaptic eCB release, we sparsely expressed α-Syn in the striatum of Syn-tKO mice as before but included mutations in the α-Syn rescue sequence to determine which regions (and, therefore, functions) of α-Syn are required for eCB-dependent plasticity. Although we previously observed that C-terminal truncation of α-Syn had no effect on eCB-LTD (Fig. 4d), we asked if C-terminal serine 129, a site previously implicated in Ca^2+^-binding affinity and regulating PD neurodegeneration^37,38^, could modulate eCB-LTD. However, we found that phosphorylation at serine 129 was not relevant for α-Syn’s function within eCB-LTD, as neither alanine (S129A, phosphorylation-deficient) nor aspartate (S129D, phosphorylation-mimic) substitutions^37,38^ affected the viral rescue of eCB-LTD in Syn-tKO mice (Extended Data Fig. 5).

**Fig. 5:**
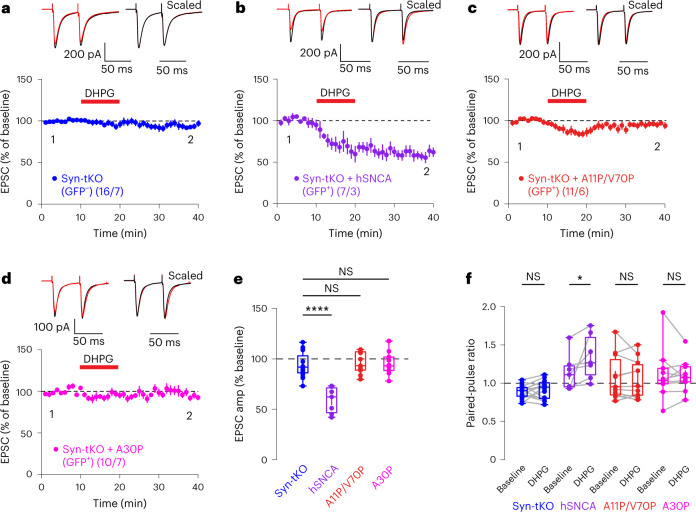
α-Syn membrane interaction domain is required for eCB-LTD. **a**–**f**, eCB-LTD is absent in GFP^−^ SPNs (**a**), rescued by full-length human α-Syn (**b**) but not rescued by the expression of mutant A11P/V70P α-Syn (**c**) or by α-Syn harboring the PD mutation A30P (**d**) (GFP^−^, pooled: *n* = 16 cells, 7 mice, 93.84 ± 3.06%; GFP^+^, hSNCA: *n* = 7 cells, 3 mice, 59.08 ± 4.90%, *P* = 4.98 × 10^−7^; A11P/V70P: *n* = 11 cells, 6 mice, 95.40 ± 3.17%; *P* = 0.986; A30P: *n* = 10 cells, 7 mice, 95.75 ± 3.59%; *P* = 0.977). **e**, Summary of EPSC amplitudes. **f**, Summary of PPRs for Syn-tKO cells infected with α-Syn (GFP^+^, hSNCA baseline: 1.14 ± 0.09; post-DHPG: 1.35 ± 0.11; *P* = 0.016; GFP^−^, pooled baseline: 0.89 ± 0.02; post-DHPG: 0.92 ± 0.03; *P* = 0.255; A11P/V70P baseline: 1.07 ± 0.09; post-DHPG: 1.05 ± 0.08; *P* = 0.496; A30P baseline: 1.12 ± 0.11; post-DHPG: 1.10 ± 0.07; *P* = 0.695). Data are mean ± s.e.m. **e**,**f**, Box plots are depicted as mean (center), first/third quartiles (lower/upper box limits) and minima/maxima (bottom/top whiskers). Statistical significance was assessed by two-sided tests, including ANOVA with multiple comparisons (**e**) and Wilcoxon signed test (**f**) (****P < 0.0001; *P < 0.05; NS, not significant). Source data

Our results, thus, indicate that the N-terminal domain of α-Syn is required for eCB release. The major biochemical activity of α-Syn consists of phospholipid membrane binding that is mediated by its N-terminal domain^6,39^. To test whether membrane binding by α-Syn is required for eCB-LTD, we virally expressed α-Syn mutants carrying A11P and V70P (A11P/V70P) substitutions that ablate membrane binding by α-Syn but do not impair its synaptic localization^6^. Remarkably, A11P/V70P mutant α-Syn failed to rescue eCB-LTD in Syn-tKO mice (Fig. 5a–c), suggesting that membrane binding of α-Syn is required for eCB-LTD. To strengthen this hypothesis, we repeated these experiments in cells infected with A30P mutant α-Syn, a PD mutation that also decreases lipid binding by α-Syn^6^. A30P mutant α-Syn also did not rescue the loss of eCB-LTD in Syn-tKO mice (Fig. 5d,e). Correspondingly, PPRs were increased in cells expressing WT α-Syn but not in cells expressing A11P/V70P mutant or A30P mutant α-Syn (Fig. 5f). Together, these results demonstrate that, in postsynaptic neurons, α-Syn enables eCB-LTD by binding to phospholipid membranes in a process that likely mediates the postsynaptic release of eCBs.

### Postsynaptic SNAREs are required for eCB release

α-Syn has been shown to act as a SNARE chaperone that facilitates SNARE complex assembly during vesicular exocytosis by binding to phospholipid membranes^14,40^. SNARE proteins not only mediate presynaptic vesicle exocytosis but are also essential for postsynaptic exocytosis of AMPA receptors and other proteins^41,42^. Thus, the fact that eCB release requires postsynaptic α-Syn that is competent to bind to phospholipid membranes suggests that eCBs are likely released by a synuclein-dependent mechanism that engages the postsynaptic membrane. To investigate this possibility, we tested if postsynaptic SNAREs are involved in eCB-dependent plasticity and eCB release.

**Fig. 6:**
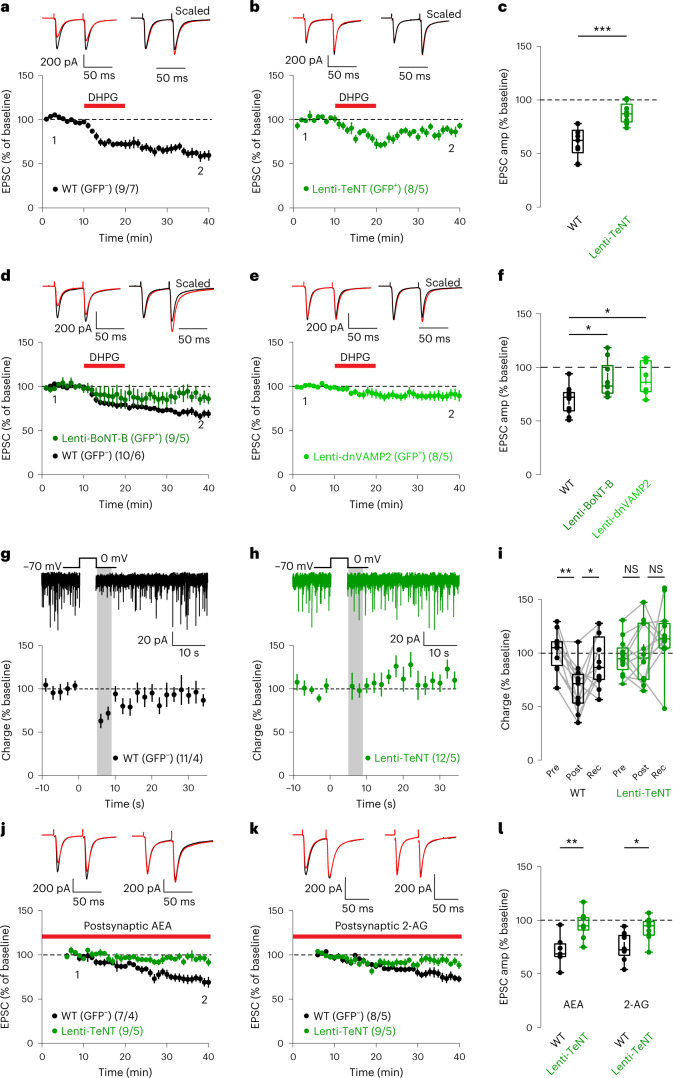
Postsynaptic SNARE function is required for eCB plasticity and eCB release. **a**–**c**, Postsynaptic lentiviral expression of TeNT impairs eCB-LTD expression (GFP^−^: *n* = 9 cells, 7 mice, 60.47 ± 4.73; GFP^+^: *n* = 8 cells, 5 mice, 87.54 ± 3.51%; *P* = 9.87 × 10^−4^). **d**–**f**, Postsynaptic lentiviral expression of BoNT-B (**d**) or dnVAMP2 (**e**) impairs eCB-LTD (GFP^−^: *n* = 10 cells, 6 mice, 69.38 ± 4.17%; BoNT-B GFP^+^: *n* = 9 cells, 5 mice, 89.14 ± 5.52%; *P* = 0.024; dnVAMP2 GFP^+^: *n* = 8 cells, 5 mice, 89.67 ± 5.58%; *P* = 0.024). **f**, Summary of EPSC amplitudes. **g**–**i**, Postsynaptic TeNT impairs striatal DSI (GFP^−^: *n* = 11 cells, 4 mice, pre-depol: 101.93 ± 5.38%, post-depol: 67.33 ± 6.50%, recovery: 91.48 ± 7.40%, *P* = 9.77 × 10^−4^, *P* = 0.032; GFP^+^: *n* = 12 cells, 5 mice, pre-depol: 97.40 ± 4.24%, post-depol: 99.58 ± 7.00%, recovery: 111.00 ± 6.81%, *P* = 0.569, *P* = 0.339). **j**–**l**, Postsynaptic TeNT prevents LTD induced by AEA loading or 2-AG loading (AEA GFP^−^: *n* = 7 cells, 4 mice, 71.57 ± 5.20%; AEA GFP^+^: *n* = 9 cells, 5 mice, 96.06 ± 4.05%, *P* = 5.2 × 10^−3^; 2-AG GFP^−^ data from Fig. 2g: *n* = 8 cells, 5 mice, 74.92 ± 4.66%; 2-AG GFP^+^: *n* = 9 cells, 5 mice, 92.01 ± 3.57%; *P* = 0.015). **l**, Summary of EPSC amplitudes. Data are mean ± s.e.m. **c**,**f**,**i**,**l**, Box plots are depicted as mean (center), first/third quartiles (lower/upper box limits) and minima/maxima (bottom/top whiskers). Statistical significance was assessed by two-sided tests, including Mann–Whitney tests (**c,l**) and Wilcoxon signed tests (**f**) (***P < 0.001; **P < 0.01; *P < 0.05; NS, not significant). depol, depolarization; Rec, recovery. Source data

**Extended Data Fig. 6:**
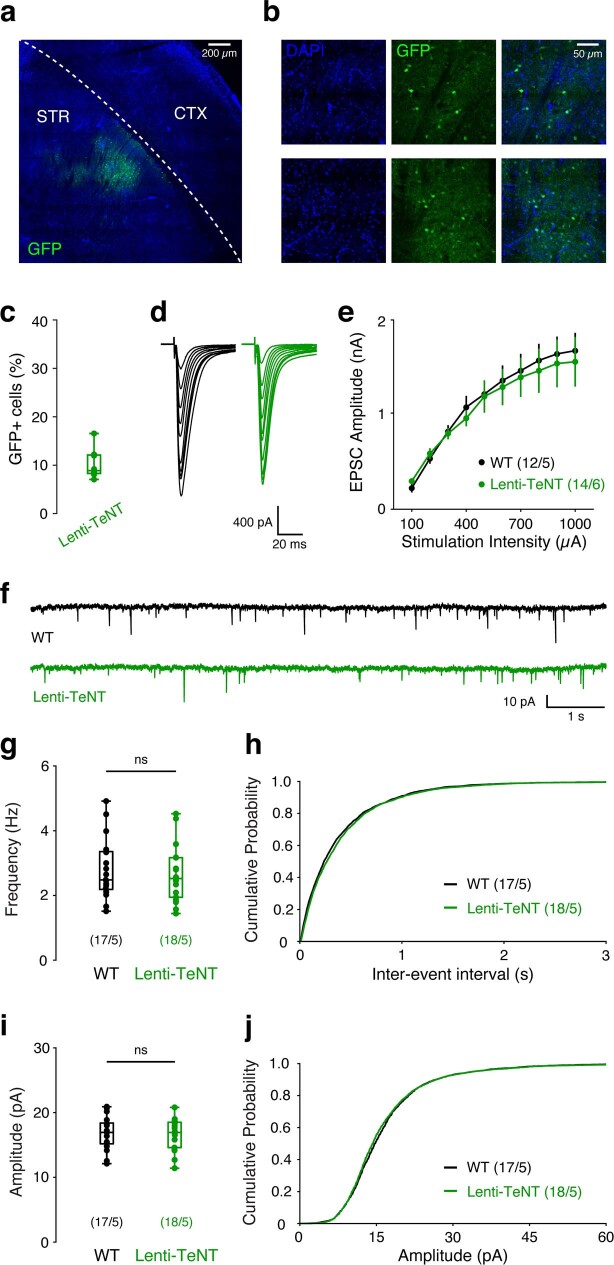
Sparse lentiviral expression of TeNT in striatal SPNs does not disrupt the basal functional properties of afferent synapses formed onto SPNs. **a**, Representative image showing lentiviral expression of GFP-2A-TeNT in the striatum (1 of 8 slices). **b**, High magnification images. Top: DAPI, middle: GFP, bottom: overlay. **c**, Quantification of infection efficiency (GFP + / DAPI+): 10.28 ± 1.10% cells, n = 8 slices / 4 mice. **d**, Representative traces of evoked corticostriatal EPSCs in WT (GFP-) and TeNT-infected (GFP+) SPNs across a range of stimulation intensities. **e**, Input-output curves (GFP-: n = 12 cells / 5 mice; GFP+: n = 14 cells / 6 mice; p = 0.787). **f**, Representative traces of mEPSC recordings from WT (GFP-) and TeNT-expressing (GFP+) cells. **g-j**, Summary of mEPSC frequency (GFP-: n = 17 cells / 5 mice, 2.81 ± 0.22 Hz; GFP+: n = 18 cells / 5 mice, 2.64 ± 0.20 Hz; p = 0.680). **h**, Cumulative distribution of mEPSC inter-event intervals. **i**, Summary of mEPSC amplitude (GFP-: 16.78 ± 0.62 pA; GFP+: 16.65 ± 0.55 pA; p = 0.987). **j**, Cumulative distribution of mEPSC amplitudes. Data are mean ± SEM. (**c, g, i**) Box plots are depicted as mean (center), first/third quartile (lower/upper box limits), and minima/maxima (bottom/top whiskers). Statistical significance was assessed by two-sided tests, including 2-way repeated measures ANOVA (**e**) and Mann-Whitney tests (**g, i**) (n.s. non-significant). Source data

**Extended Data Fig. 7:**
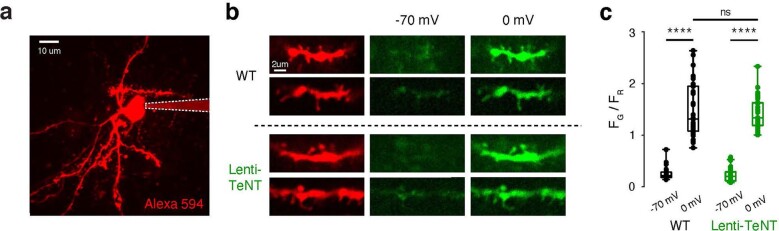
2-photon Ca^2+^ imaging in SPN dendrites shows that lentiviral expression of TeNT does not alter SPN dendritic Ca^2+^ signaling. **a**, Representative 2-photon image of a SPN (1 of 8 cells). **b**, Images of dendritic segments in red (Alexa594, 50 µM) and green (Fluo-5F, 300 µM) channels while cells were depolarized from -70 mV to 0 mV from WT (top) and TeNT-expressing (bottom) SPNs. **c**, Summary of depolarization-induced changes in dendritic Ca^2+^ (GFP-, n = 33 dendrites / 4 cells / 3 mice, -70 mV: 0.25 ± 0.11, 0 mV: 1.45 ± 0.53; p = 3.77e-9; GFP + / TeNT-expressing SPNs, n = 36 dendrites / 4 cells / 3 mice, -70 mV: 0.23 ± 0.14, 0 mV: 1.42 ± 0.30; p = 3.77e-9; GFP- vs. GFP+, p = 0.84). Data are mean ± SEM. (**c**) Box plots are depicted as mean (center), first/third quartile (lower/upper box limits), and minima/maxima (bottom/top whiskers). Statistical significance was assessed by two-sided ANOVA with multiple comparisons (**c**) (**** p < 0.0001; n.s. non-significant). Source data

**Extended Data Fig. 8:**
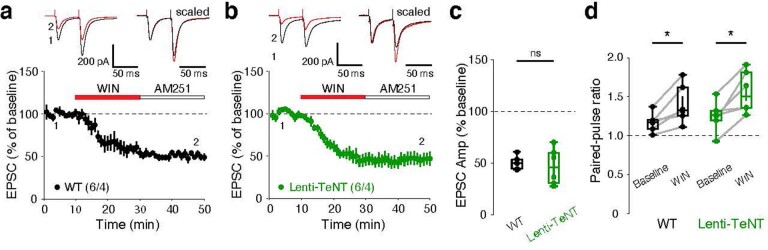
Presynaptic CB1R function is not disrupted by lentiviral TeNT expression. **a, b**, WIN-induced corticostriatal LTD in GFP- (**a**) and GFP + (TeNT-expressing) (**b**) SPNs. **c, d**, Summary of EPSC amplitudes (**c**) (GFP-: n = 6 cells / 4 mice, 50.36 ± 2.59%; GFP+: n = 6 cells / 4 mice, 46.73 ± 7.16%; p = 0.818) and of PPRs (**d**) in GFP- (baseline: 1.17 ± 0.05; post-WIN: 1.40 ± 0.10; p = 0.031) and GFP + SPNs (baseline: 1.25 ± 0.08; post-WIN: 1.56 ± 0.11; p = 0.031). Data are mean ± SEM. (**c, d**) Box plots are depicted as mean (center), first/third quartile (lower/upper box limits), and minima/maxima (bottom/top whiskers). Statistical significance was assessed by two-sided tests, including Mann-Whitney tests (**c**) and Wilcoxon signed tests (**d**) (* p < 0.05; n.s. non-significant). Source data

**Extended Data Fig. 9:**
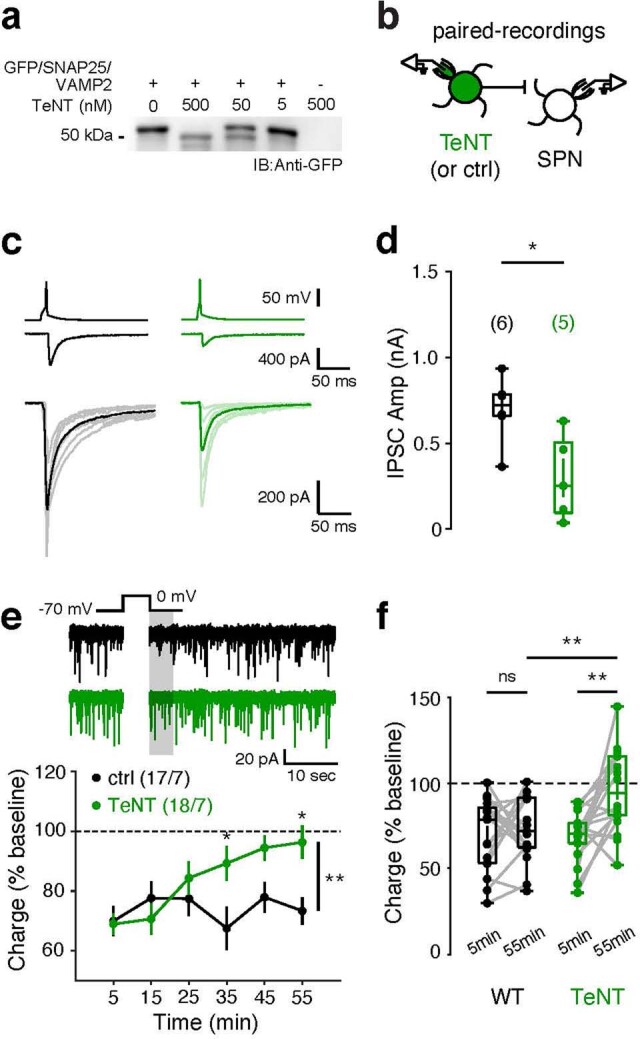
Acute loading of recombinant TeNT-LC through the patch pipette disrupts DSI in striatal SPNs. **a**, Western blot image showing Synaptobrevin-2/VAMP2 cleavage in the presence of recombinant TeNT-LC protein dissolved in recording internal solution (conducted once before all recording experiments). **b**, Schematic of paired recordings. **c**, Top: representative paired recordings showing monosynaptic IPSCs in response to evoked presynaptic action potentials. Bottom: Overlay of time-aligned monosynaptic IPSCs across recorded cells. **d**, Summary of IPSC amplitudes recorded from postsynaptic SPNs. Acute presynaptic dialysis of TeNT (~20 mins) is sufficient to significantly reduce monosynaptic IPSC amplitudes (ctrl: n = 6 pairs / 4 mice, 697.62 ± 78.34 pA; TeNT: 5 pairs / 4 mice, 299.07 ± 109.81 pA; p = 0.017). **e, f**, Acute TeNT-loading in WT SPNs progressively disrupts striatal DSI (ctrl: 17 cells / 7 mice, 5 min: 69.94 ± 5.11%, 55 min: 73.35 ± 4.56%; ctrl 5 min vs. 55 min: p = 0.554; TeNT: 18 cells / 7 mice, 5 min: 69.00 ± 3.54%, 55 min: 96.37 ± 5.59%; TeNT 5 min vs. 55 min: p = 0.003; ctrl 55 min vs. TeNT 55 min: p = 0.008; ctrl vs. TeNT: p = 0.005). Top, representative DSI recordings. **f**, Summary of DSI at 5 min (left) and 55 min (right) after TeNT-loading. Data are mean ± SEM. (**d, f**) Box plots are depicted as mean (center), first/third quartile (lower/upper box limits), and minima/maxima (bottom/top whiskers). Statistical significance was assessed by two-sided tests, including Mann-Whitney tests (**d**), 2-way repeated measures ANOVA (**E**), and ANOVA with multiple comparisons (**f**) (** p < 0.01; * p < 0.05; n.s. non-significant). Source data

We sparsely infected SPNs in the dorsolateral striatum of WT mice with lentiviruses that co-express GFP and tetanus toxin light chain (TeNT), which inactivates synaptobrevin-2, a SNARE protein involved in most forms of exocytosis. We confirmed that postsynaptic TeNT expression did not disrupt basal synaptic properties of infected SPNs, as previously shown for hippocampal neurons^43^ (Extended Data Fig. 6). Next, we measured eCB-dependent plasticity, comparing GFP^+^ (TeNT-expressing) cells to adjacent uninfected GFP^−^ controls. Strikingly, TeNT significantly impaired eCB-LTD (Fig. 6a–c). Notably, TeNT expression did not disrupt postsynaptic dendritic excitability (Extended Data Fig. 7) or presynaptic CB1R function (Extended Data Fig. 8). Additionally, we found that lentiviral expression of botulinum toxin type B light chain (BoNT-B) or of a dominant-negative form of synaptobrevin-2 (dnVAMP2)^44,45^ also significantly disrupted eCB-LTD (Fig. 6d–f), demonstrating the requirement of postsynaptic SNAREs for normal eCB-LTD. Notably, lentiviral TeNT also blocked striatal DSI (Fig. 6g–i), an effect that was not revealed in previous studies using acute neurotoxin dialysis through the patch-clamp recording pipette^25^. We revisited this experiment by loading postsynaptic SPNs with recombinant TeNT light chain protein^44^. Acute dialysis of TeNT progressively disrupted striatal DSI (Extended Data Fig. 9). Together, the impaired eCB-LTD and DSI results mirror the Syn-tKO phenotypes and suggest that postsynaptic SNAREs are required for eCB-dependent plasticity. Lastly, to further explore the specificity of the effect of TeNT in impairing the release of eCBs, we performed the AEA-loading and 2-AG-loading experiment as before. AEA loading or 2-AG loading of GFP^+^ cells expressing TeNT failed to induce progressive synaptic depression, whereas loading of GFP^−^ control cells robustly suppressed synaptic transmission (Fig. 6j–l). Thus, in addition to synucleins, SNAREs are required postsynaptically for the active release of eCBs, suggesting that they collaborate in the postsynaptic release and/or transport of eCBs.

## Discussion

Here we show that eCBs are released by a postsynaptic process that requires synucleins and SNAREs. Thus, we report an unexpected convergence of two puzzling questions in neuroscience, namely the questions of the function of synucleins and of the mechanism of eCB release. We show that mice lacking all three synuclein isoforms have apparently normal basal synaptic properties but exhibit significant defects in multiple forms of eCB-dependent plasticity spanning different timeframes (eCB-LTD and DSI), synapse types (glutamatergic and GABAergic) and brain regions (striatum and hippocampus). Using direct measurements of eCB release, we demonstrate that synuclein-deficient neurons suffer from a loss of eCB release but retain normal CB1R function. Strikingly, bypassing the Ca^2+^-dependent eCB synthesis processes via postsynaptic loading of neurons with AEA or 2-AG (endogenous eCBs) revealed that the secretion of eCBs from the postsynaptic cell, and not their synthesis, is impaired by the synuclein deletion. Consistent with this conclusion, AEA and 2-AG levels are not impaired by the synuclein deletion (Extended Data Fig. 4e,f). Mechanistically, we identify the N-terminal membrane-binding domain of α-Syn (Fig. 5), as well as postsynaptic synaptobrevin SNAREs, as required for eCB release.

Together, these results perhaps suggest that vesicular exocytosis is involved in the release of eCBs, or, alternatively, postsynaptic synucleins may be involved in a SNARE-dependent, trans-membrane transport process that is yet to be identified. For example, synucleins may be released themselves via exosomes in a SNARE-dependent process together with eCBs, although the question arises of how exosomal eCBs would interact with presynaptic CB1Rs^46–48^. Previous work has shown that α-Syn has great affinity for membrane curvature, including synaptic vesicles, where it can regulate the biophysics of exocytosis and vesicular recycling^3^. Thus, it is possible that postsynaptic α-Syn is involved in a membrane interaction process that directly facilitates the transport of eCBs across the membrane or helps assemble the required carriers to mediate eCB transport, such as FABP5 and/or extracellular vesicles^46^. Furthermore, our findings that postsynaptic SNAREs are simultaneously required for eCB release are consistent with reports showing α-Syn’s role in organizing presynaptic SNARE complexes^14,40^ and the growing evidence that postsynaptic SNAREs are involved in regulating synaptic plasticity^42^.

Our results are surprising given that α-Syn is canonically regarded as functioning presynaptically and, indeed, do not preclude additional presynaptic roles for α-Syn. Our viral α-Syn rescue experiments take advantage of the corticostriatal circuit’s compartmentalization of presynaptic and postsynaptic cells to demonstrate that the selective postsynaptic expression of α-Syn is sufficient to restore eCB-dependent plasticity in Syn-tKO mice. Various reports have suggested that presynaptic α-Syn modulates transmitter release in a use-dependent manner, as repeated stimulation is often required to reveal its effect on exocytosis^10,11^. Combined with our novel findings that demonstrate a postsynaptic role for synucleins in inhibiting synaptic transmission in a plasticity context, a broader role for synucleins emerges as activity-dependent regulators of synaptic transmission, operating from both sides of the synapse.

Our results are also unexpected because a few reports have suggested that SNAREs are not involved in eCB release^24,25^. However, it is important to note that these previous studies relied on acute botulinum toxin light chain dialysis, which may be temporally insufficient to block eCB release, as clostridial neurotoxins cannot cleave preassembled SNARE complexes^49^. Indeed, there are many cases where acute clostridial neurotoxins are unable to block SNARE-dependent vesicular release^50,51^. In our study, we achieved neurotoxin expression (for example, Lenti-GFP-TeNT) that enables TeNT action for multiple days before experiments, allowing for more complete SNARE cleavage. In any case, it is also worth noting that a SNARE-dependent, vesicular process does not necessarily point toward a traditional vesicular exocytosis mechanism^46–48^. Notably, our viral rescue experiments used neuron-specific expression of various forms of α-Syn, suggesting a neuronal mechanism, but our results do not rule out an additional involvement of glia, which may very well contribute to the overall process^52–54^.

Finally, our results provide a potential link between eCB signaling and PD. Significant bidirectional signaling has been described between the eCB system and the dopaminergic system^30,55^. Striatal SPNs express CB1Rs and DA receptors, and eCB-dependent plasticity in the direct and indirect pathways is modulated by DA signaling^30^. Conversely, DA neurons synthesize and release eCBs, and many of their inputs and local midbrain circuitry express CB1Rs^55,56^. Genetic or pharmacological manipulation of eCB signaling alters striatal DA levels^57^. Furthermore, eCB plasticity is significantly impaired in DA depletion models of PD^19,32,58^, which may contribute to the striatal hyperactivity and subsequent cortical hypoactivity observed in PD. In particular, our finding that A30P PD mutant α-Syn is unable to rescue eCB-LTD suggests that eCB release and eCB-dependent plasticity may be aberrant in PD, potentially contributing to the cognitive deficits observed in patients with PD. This notion is attractive given the availability of potent pharmaceutical agents acting on CB1Rs, but it requires further validation.

Together, our results demonstrate an unexpected postsynaptic function of endogenous synucleins in regulating eCB release and synaptic plasticity, which identifies a biological process that is completely dependent on synucleins. Whether synucleins’ role in eCB release is related to their function in preventing neurodegeneration caused by the deletion of CSPα^15^ remains to be clarified. Given that our results also reveal that eCBs are likely released via a synuclein-dependent postsynaptic membrane mechanism, they reconcile two open questions in neuroscience—namely, how eCBs are released and for what functions synucleins are essential— thereby forming the basis for further insights into the modulatory mechanisms that control neural circuits in healthy and neurodegenerative brains.

## Experimental model and subject details

### Animals

All experiments were performed in accordance with protocols approved by the Stanford University Animal Care and Use Committee in keeping with the National Institutes of Health’s *Guide for the Care and Use of Laboratory Animals*. Animals were kept at a 12-h light/dark cycle at a room temperature of 22 °C with humidity control (30–70%). Both male and female mice were used for all experiments at ~3 months old (P70–P100), with the exception of recordings from aged mice (16–18 months old). Syn-tKO mice (α-Syn^−/−^;β-Syn^−/−^;γ-Syn^−/−^) were generated as previously described^28^. WT C57BL/6 mice were maintained as controls, and Syn-tKO mice were backcrossed to C57BL/6 every 6–10 months to maintain a consistent background between Syn-tKO and WT lines. α-Syn-KO (α-Syn^−/−^) and βγ-Syn-KO (β-Syn^−/−^;γ-Syn^−/−^) were generated from these backcrosses. Stereotaxic injections were performed 2–6 weeks before recordings.

## Methods

### Acute brain slice preparation

Adult mice (male and female) were anesthetized with isoflurane and decapitated, and brains were extracted and briefly submerged into chilled artificial cerebrospinal fluid (ACSF) containing 125 mM NaCl, 2.5 mM KCl, 1.25 mM NaH_2_PO_4_, 25 mM NaHCO_3_, 15 mM glucose, 2 mM CaCl_2_ and 1 mM MgCl_2_, oxygenated with 95% O_2_ and 5% CO_2_ (300–305 mOsm, pH 7.4). Oblique horizontal slices (300-µm thickness) containing dorsal striatum (or coronal slices containing hippocampus) were then prepared using a tissue vibratome (VT1200S, Leica), incubated in chambers containing 34 °C ACSF for 30 min and then allowed to recover at room temperature for 30 min. After recovery, slices were transferred to a submerged recording chamber perfused with ACSF at a rate of 2–3 ml min^−1^ at a temperature of 30–31 °C. All recordings were performed within 5 h of slice recovery.

### Whole-cell slice electrophysiology

Whole-cell voltage-clamp recordings were made with glass pipettes (3–4 MΩ) filled with internal solution containing 126 mM CsMeSO_3_, 10 mM HEPES, 1 mM EGTA, 2 mM QX-314 chloride, 0.1 mM CaCl_2_, 4 mM Mg-ATP, 0.3 mM Na_3_-GTP and 8 mM disodium phosphocreatine (280–290 mOsm, pH 7.3 with CsOH), and cells were voltage clamped at −70 mV unless specified otherwise. Access resistance was measured by injection of hyperpolarizing pulses (−5 mV, 100 µs) and was less than 25 MΩ for all recordings (<35 MΩ for recombinant TeNT-loading experiments), and only cells with a change in access resistance <20% throughout the entire experiment were included in the analysis. Similarly, input resistance was monitored throughout the entirety of experimental recordings. For EPSC recordings, 50 µM picrotoxin was added to block GABA_A_ receptor-mediated currents. Evoked EPSCs were elicited by stimulating axons via a concentric bipolar stimulating electrode (FHC). Whole-cell patch-clamp recordings were performed using a MultiClamp 700B (Molecular Devices), monitored with WinWCP (Strathclyde Electrophysiology Software) and analyzed offline using Clampfit 10.0 (Molecular Devices) and custom-made MATLAB (Mathworks, 2020b) software. Signals were filtered at 2 kHz and digitized at 10 kHz (NI PCIe-6259, National Instruments).

### Basal corticostriatal synaptic activity recordings

For input–output curves of corticostriatal synapses, three EPSCs were averaged at stimulation intensities ranging from 100 µA to 1,000 µA (100-µA step size), and the average amplitude was measured. For measuring dynamics of repeated stimulation, trains of 40 stimulation pulses were delivered at a range of frequencies (2, 5, 10, 20, 50 and 100 Hz)^13^. Miniature excitatory postsynaptic currents (mEPSCs) were measured by continuously recording for 10 min in the presence of 1 µM tetrodotoxin to prevent action potential firing and 50 µM picrotoxin to block GABA_A_ receptor-mediated currents.

### eCB-LTD recordings

For long-term eCB-LTD recordings, a pair of EPSCs (50-ms interval) were evoked at 0.05 Hz, and three successive EPSCs were averaged and quantified relative to the normalized baseline. For DHPG-mediated eCB-LTD experiments, cells were slightly depolarized to −50 mV, and DHPG (50 µM) was added to the perfusion after a baseline period^19,31^. For WIN-mediated LTD experiments, WIN (2 µM) was added to the perfusion. In various control experiments, AM251 (10 µM) was added to the perfusion to block CB1Rs. PPRs were measured by dividing the peak amplitude of the second evoked EPSC by the first EPSC.

### DSI recordings

For DSI experiments, a high-chloride internal solution was used including 125.2 mM CsCl, 10 mM NaCl, 10 mM HEPES, 1 mM EGTA, 2 mM QX-314 chloride, 0.1 mM CaCl_2_, 4 mM Mg-ATP, 0.3 mM Na_3_-GTP and 8 mM disodium phosphocreatine (280–290 mOsm, pH 7.3 with CsOH). NBQX (10 µM) and R-CPP (10 µM) were included in the perfusion to block AMPAR-mediated and NMDAR-mediated currents, respectively. In DSI experiments measuring sIPSC charge, high-Ca^2+^ ACSF was used (4 mM Ca^2+^, 0.5 mM Mg^2+^) to increase the rate of spontaneous events, and sIPSCs were recorded for a baseline of 60 s before depolarization to 0 mV for 5 s and additional recording of sIPSCs for 60 s after depolarization^25^. sIPSC charge (integrated current) was binned every 2 s, normalized to the average of the 10 s (five bins) preceding depolarization, and the normalized charge before depolarization, after depolarization and 20 s after depolarization were compared. In DSI experiments using pipette loading of recombinant TeNT (Extended Data Fig. 9), DSI was induced every 2 min from *t* = 5 min to *t* = 60 min relative to whole-cell break-in, with 3–5 DSI values averaged within each 10-min bin. For DSI experiments measuring evoked IPSCs, a pair of evoked IPSCs (50-ms interval) were evoked at 0.2 Hz, and average peak amplitude and average PPR were measured before, after and 20 s after depolarization. Three traces were averaged per cell.

### AEA-loading and 2-AG-loading LTD recordings

For AEA-loading and 2-AG-loading experiments, AEA (50 µM) or 2-AG (50 µM) was included in the internal solution as previously described^35^. In brief, evoked EPSCs were recorded starting 5 min after achieving whole-cell configuration to allow EPSC amplitudes to stabilize. Baseline periods were measured in the 5–10-min period after whole-cell break-in, and all peak amplitudes were normalized to the average EPSC amplitude during this baseline period.

### Viral plasmid construction

To generate pAAV-hSyn-GFP-IRES-mSNCA/hSNCA plasmids, GFP, IRES and SNCA coding sequences were cloned and sequentially stitched using overlapping polymerase chain reaction (PCR). Then, GFP-IRES-mSNCA/hSNCA fragments were digested with *Age*I/*Nhe*I and inserted into a pAAV-hSyn-Empty plasmid (Ding laboratory collection). Specifically, hSNCA and mSNCA were amplificated using pTB-hSyn-hSNCA and pTB-hSyn-mSNCA plasmids (gifts from the Südhof laboratory) as templates, respectively. To truncate mSNCA, a pair of primers were used to amplificate the coding sequence of 1–95 amino acids of the mSNCA. Then, full-length mSNCA was removed by XbaI/NheI digestion and replaced by mSNCA (1–95) to generate the pAAV-hSyn-GFP-IRES-mSNCA (1–95) plasmid. Similarly, we introduced S129A, S129D, A11P/V70P or A30P mutations into pAAV-hSyn-GFP-IRES-hSNCA construct by replacing the WT hSNCA with corresponding mutants. All mutants were subcloned from pCMV5-hSNCA mutant plasmids (gifts from the Südhof laboratory). For the TeNT lentivirus, a FUW-UBC-EGFP-2A-TeNT plasmid (gift from the Südhof laboratory) was used. For BoNT-B and dnVAMP2 plasmids, the BoNT-B sequence was amplified from a previous plasmid (gift from the Südhof laboratory) using PCR, and the dnVAMP2 sequence (corresponding sequence of 1–96 amino acids of VAMP2) was synthesized, after which each sequence was subcloned into the FUW-UBC-EGFP-2A-X plasmid to generate the BoNT-B and dnVAMP2 viral plasmids. For viral packaging, all plasmids were prepared using EndoFree Plasmid Maxi Kit (Qiagen, 12362).

### Viral packaging

All SNCA viruses were packaged into AAV8 capsid and purified by discontinuous iodixanol gradients and ultracentrifugation as previously described^59^. In brief, 640 µl (1 mg ml^−1^) of polyethylenimine hydrochloride (PEI) solution (MW 40 kDa, pH 7.0, cat. no. 24765) was mixed with serum-free DMEM media containing 35 µg of AAV genome plasmid, 35 µg of AAV8 capsid plasmid (AAV8-Rep/Cap) and 100 µg of helper plasmid (pHGTI-adeno1) and incubated at room temperature for 15 min. Then, DNA/PEI mixture was slowly added into 293T cell culture (5 × 15-cm dishes) and mixed well. After incubation with 293T cells at 37 °C for 24 h, transfection media was replaced with fresh serum-free DMEM. Seventy-two hours after transfection, culture media was harvested and filtered through 0.44-µm filters to get rid of cells and debris. To precipitated virus, collected media was incubated with 0.4 M NaCl and 8.5% PEG8000 at 4 °C for 1.5 h, followed by spinning down at 7,000*g* for 10 min. Viral particles were resuspended with 10 ml of lysis buffer (150 mM NaCl, 20 mM Tris, 10 mM MgCl_2_, pH 8.0) and then incubated with 25 U ml^−1^ benzonase (Sigma-Aldrich, E8263) at 37 °C for 10 min. Crude virus isolate was then transferred to the top layer of a iodixanol step gradient (15%, 25%, 40% and 60%) and centrifugated at 190,000*g* (Beckman VTi50 rotor) for 90 min at 4 °C. Purified viruses were collected from a new formed layer between 40% and 60% layers after centrifugation, washed twice with PBS and concentrated using Amicon Ultra-15 centrifugal filter units (100 kDa, EMD Millipore, UFC10008). Viruses were aliquoted and stored at −80 °C. Then, 5 µl of virus was resolved by SDS-PAGE gel for purity assessment and semi-quantitative titration. All AAV construct titers were in the ~10^13^–10^14^ particles per milliliter range. TeNT, BoNT-B and dnVAMP2 lentiviruses were prepared by the Stanford Gene Vector and Virus Core, with IU ml^−1^ of 1.10 × 10^9^, 1.51 × 10^9^ and 1.09 × 10^9^, respectively. Sequences of all viral constructs can be found in Supplementary Table 1.

### Stereotaxic viral injections

Stereotaxic injections of AAVs and lentiviruses were performed on male and female adult mice (3 months old) under isoflurane anesthesia. A total volume of 100–300 nl was injected unilaterally into the left dorsal striatum (from bregma, AP: 1.0, ML: 2.4, DV: 3.4). Injections were performed using a micropipette (VWR) pulled with a long, narrow tip size (~10–20 µm) using a micropipette puller (Sutter Instrument). Glass micropipettes were slowly inserted into the brain and left for 10 min before virus was injected at an infusion rate of 100 nl min^−1^. The pipettes were then slowly retracted 10 min after infusion, and animals were sutured and monitored after surgery. Acute brain slice recordings were performed 2–6 weeks after injections, where infected cells were identified by GFP fluorescence (BX51, Olympus).

### Two-photon imaging of GRAB_eCB2.0_

Four weeks after stereotaxic injection (see above) of AAV9-hSyn-GRAB_eCB2.0_ (ref. ^56^), acute brain slices were prepared (see above) for imaging. Two-photon imaging was performed using a custom-modified scanning microscope controlled with ScanImage and equipped with a mode-locked tunable Mai Tai Ti:sapphire laser (Spectra-Physics) with a low laser power (output optical power <40 mW) to avoid phototoxicity, and a ×60/1.1 NA water-immersion objective. A 920-nm wavelength was used to excite the GRAB_eCB2.0_ sensor, and fluorescence was collected using a 495–540-nm filter. Electrical stimulation consisted of 10 pulses (0.2-ms duration) delivered at 20 Hz^36^. Pharmacological experiments included addition of 10 µM AEA and/or 10 µM AM251 to the ACSF perfusion at 2–3 ml min^−1^. All images were acquired at a frame rate of 2 Hz with a resolution of 512 × 512 pixels. The average pixel intensity of each frame was quantified and normalized to the baseline intensity (average intensity of first four frames (2 s) before stimulation) to quantify GRAB_eCB2.0_ sensor activity and response to pharmacology. Imaging data were analyzed using ImageJ software and custom MATLAB (MathWorks, 2020b) code.

### Two-photon imaging of dendritic Ca^2+^ activity

Ten days after stereotaxic injection (see above) of lenti-TeNT, acute brain slices were prepared (see above) from young adult (~3 months old) mice. Two-photon imaging was performed (see above) during whole-cell patch-clamp of SPNs filled with both Alexa Fluor 594 (50 µM) and Fluo-5F (300 µM). After ~20 min of internal solution dialysis, SPN dendrite segments (~80–120 µm from soma) were imaged using a wavelength of 830 nm (exciting both Alexa Fluor 594 and Fluo-5F). Red and green channels were collected at −70 mV for 5 s (2-Hz frame scan) and 5 s after depolarization to 0 mV. Manual regions of interest (ROIs) of dendritic segments were then quantified using the average green channel signal (F_G_) / red channel signal (F_R_) at −70 mV and 0 mV.

### Measurement of AEA and 2-AG

Acute brain slices were prepared (see above) from young adult (~3 months old) mice. Three coronal sections (300 µm each) per brain (~0.6–1.5 anterior to bregma) (tissue weight ~58 mg) were collected 1 h after slice recovery. Tissue was then homogenized in PBS (tissue weight (g): PBS (ml) volume = 1:9) using a Dounce grinder on ice and sonicated for 30 s with an ultrasonic cell disrupter. The homogenates were centrifuged for 30 min at 20,000*g*, and the supernatants were collected for measuring AEA and 2-AG concentrations with custom ELISA kits (MyBioSource) according to the manufacturer’s instructions. All samples were measured at 450 nm in duplicate, and their concentrations were calculated using the exponential equation that best fits the standard curve.

### Immunocytochemistry

In a subset of recordings, the brain slices were fixed by transferring to wells of 4% paraformaldehyde in 0.1 M phosphate buffer (0.1 M PB, pH 7.4) overnight at 4 °C. Slices were then washed in PBS three times (10 min each) at room temperature before being mounted using an antifade mounting medium including a nuclear DAPI stain (Vector Laboratories). For α-Syn staining experiments (for example, Figure 4), fixed slices were washed in PBS three times (10 min each) before undergoing a block incubation with 2% BSA and 10% normal donkey serum in PBS with 0.5% Triton X-100 (Sigma-Aldrich, PBS-T) (1 h, room temperature) to reduce non-specific binding. Slices were then incubated in a primary antibody solution containing antibodies against α-Syn (mouse IgG1, 1:1,000 dilution, BD Biosciences, 610786) and GFP (goat IgG, 1:100, Abcam, ab5450) overnight at 4 °C, followed by secondary antibodies conjugated to Alexa Fluor 647 (anti-mouse, 1:2,000, Thermo Fisher Scientific, A32728), Alexa Fluor 488 (anti-goat, 1:2,000, Invitrogen, A-11055) and Alexa Fluor 555 (for biocytin-filled cells) (streptavidin, 1:1,000, Invitrogen, S32355) (1 h, room temperature) before washing and mounting. Images were acquired using a confocal microscope (Leica, DM2500) with consistent settings used across all slices.

### TeNT cleavage assay

Recombinant TeNT light chain protein (R&D Systems, 6535-ZN) was dissolved in recording internal at various concentrations along with recombinant GFP/SNAP25B/VAMP-2 (R&D Systems, 7375-SV). Quality control and purification specifications were provided by R&D Systems (we did not perform additional verification of these data). Samples were incubated at 31 °C for 1 h, after which 10 µl of TeNT cleaved and control samples were resolved with a 10% SDS-PAGE gel (Bio-Rad, 1610738) in Tris/SDS/glycine buffer (Bio-Rad, 1610732) at 150 V for 2 h and then transferred to PVDF membranes using a Trans-Blot Turbo System (Bio-Rad, 1704150EDU). PVDF membranes were blocked with 5% non-fat milk/TBS and incubated with anti-GFP antibody (1:1,000, Santa Cruz Biotechnology, sc-9996) and anti-mouse IgG HRP antibody (1:5,000, Santa Cruz Biotechnology, SC-516102-CM). Chemiluminescent signals were generated by an ECL kit (Pierce, 32109) and recorded with a AI600 imaging system (GE Healthcare).

### Paired whole-cell recordings

Acute brain slices of the dorsolateral striatum were prepared from young adult mice (~3 months old). Pairs of SPNs were recorded (<60-µm distance), where putative presynaptic SPNs were loaded with recombinant TeNT light chain protein (R&D Systems, 6535-ZN) or control buffer, dissolved in a K^+^-based internal solution (135 mM KMeSO_3_, 8.1 mM KCl, 10 mM HEPES, 8 mM Na_2_-phosphocreatine, 0.3 mM GTP-Na, 4 mM ATP-Mg, 0.1 mM CaCl_2_, 1 mM EGTA, pH 7.2–7.3, osmolarity 285–290 mOsm), and putative postsynaptic SPNs were recorded using a high-chloride internal solution (see above). Monosynaptic IPSCs were evoked using a brief current injection in presynaptic SPNs (~1–2 ms, ~1.0–1.5 nA), whereas postsynaptic cells were voltage clamped at −70 mV. Upon the confirmation of monosynaptic IPSCs, average evoked IPSC amplitudes were measured for six consecutive stimulations (>15 min after presynaptic whole-cell break-in).

### Quantifications and statistical analysis

#### Power analysis

To determine sample sizes, we used the formula N = [Z × S / E]^2^, where Z is the statistical significance level, S is the standard deviation and E is the margin of error. For example: in determining N for an LTD experiment, one could used Z = 1.96 (corresponding to *P* = 0.05), S = 6.7% (realistic standard deviation based on previous LTD experiments conducted in the laboratory) and E = 5% (we want statistical power to determine a 5% difference in LTD magnitude between samples). Thus, N = [1.96 × 0.067 / 0.05]^2^ = ~7 cells needed.

#### Statistics

Animal subjects and cell recordings were randomized within experimental blocks to yield equal sampling of experimental conditions. All experiments were conducted in a blinded fashion (that is, experimenter did not know the mouse genotype). Data distribution was assumed to be normal, but this was not formally tested (hence, individual data points are presented in all statistical comparisons). Repeated measurements (for example, input–output curves and repeated stimulation release dynamics) were analyzed using two-way repeated-measures ANOVA with post hoc tests. All two-sample comparisons (for example, LTD comparisons and PPRs) were analyzed with non-parametric tests (Mann–Whitney or Wilcoxon tests). Unless otherwise specified, two-sided statistical tests were conducted, and data are presented as mean ± s.e.m., with all statistical tests, statistical significance values and sample sizes described in the figure legends. Statistical thresholds used were as follows: *P < 0.05; **P < 0.01; ***P < 0.001; ****P < 0.0001; NS, not significant.

### Lead contact and materials availability

This study did not generate new unique reagents. Further information and requests for resources and reagents should be directed to, and will be fulfilled by, the lead contacts: Thomas C. Südhof (tcs1@stanford.edu) and Jun B. Ding (dingjun@stanford.edu).

### Reporting summary

Further information on research design is available in the Nature Portfolio Reporting Summary linked to this article.

## Online content

Any methods, additional references, Nature Portfolio reporting summaries, source data, extended data, supplementary information, acknowledgements, peer review information; details of author contributions and competing interests; and statements of data and code availability are available at 10.1038/s41593-023-01345-0.

## Supplementary information

Reporting Summary

Supplementary Table 1 Supplementary Table 1: Genetic sequences for all viral plasmids used in this study.

## Data Availability

All source data are provided with this paper. Raw electrophysiology and imaging datasets are available from the corresponding authors upon reasonable request. Plasmids for our newly generated viral constructs have been deposited at https://www.addgene.org/plasmids/articles/28225278/. Source data are provided with this paper.

## Source data

Source Data Fig. 1 Statistical Source Data
Source Data Fig. 2 Statistical Source Data
Source Data Fig. 3 Statistical Source Data
Source Data Fig. 4 Statistical Source Data
Source Data Fig. 5 Statistical Source Data
Source Data Fig. 6 Statistical Source Data
Source Data Extended Data Fig. 1 Statistical Source Data
Source Data Extended Data Fig. 2 Statistical Source Data
Source Data Extended Data Fig. 3 Statistical Source Data
Source Data Extended Data Fig. 4 Statistical Source Data
Source Data Extended Data Fig. 5 Statistical Source Data
Source Data Extended Data Fig. 6 Statistical Source Data
Source Data Extended Data Fig. 7 Statistical Source Data
Source Data Extended Data Fig. 8 Statistical Source Data
Source Data Extended Data Fig. 9 Statistical Source Data
Source Data Extended Data Fig. 9 Unprocessed gel for Extended Data Fig. 9a

## Code availability

All of the code and the analyses used in this study have been deposited in a freely accessible repository at 10.5281/zenodo.7761178.
