# Supplementary material for: Postsynaptic synucleins mediate endocannabinoid signaling
Source: Nat Neurosci. 2023 May 29;26(6):997–1007. doi: 10.1038/s41593-023-01345-0 (PMC10244176; doi:10.1038/s41593-023-01345-0)
Supplement: Supplementary file 1 — Reporting Summary [file 41593_2023_1345_MOESM1_ESM.pdf]

nature portfolio

Corresponding author(s): Jun Ding

Last updated by author(s): March 24, 2023

Reporting Summary

Nature Portfolio wishes to improve the reproducibility of the work that we publish. This form provides structure for consistency and transparency in reporting. For further information on Nature Portfolio policies, see our Editorial Policies and the Editorial Policy Checklist.

Statistics

For all statistical analyses, confirm that the following items are present in the figure legend, table legend, main text, or Methods section.

n/a	Confirmed	
☐	☒	The exact sample size (n) for each experimental group/condition, given as a discrete number and unit of measurement
☐	☒	A statement on whether measurements were taken from distinct samples or whether the same sample was measured repeatedly
☐	☒	The statistical test(s) used AND whether they are one- or two-sided<br>*Only common tests should be described solely by name; describe more complex techniques in the Methods section.*
☐	☒	A description of all covariates tested
☐	☒	A description of any assumptions or corrections, such as tests of normality and adjustment for multiple comparisons
☐	☒	A full description of the statistical parameters including central tendency (e.g. means) or other basic estimates (e.g. regression coefficient) AND variation (e.g. standard deviation) or associated estimates of uncertainty (e.g. confidence intervals)
☐	☒	For null hypothesis testing, the test statistic (e.g. F, t, r) with confidence intervals, effect sizes, degrees of freedom and P value noted<br>*Give P values as exact values whenever suitable.*
☒	☐	For Bayesian analysis, information on the choice of priors and Markov chain Monte Carlo settings
☒	☐	For hierarchical and complex designs, identification of the appropriate level for tests and full reporting of outcomes
☒	☐	Estimates of effect sizes (e.g. Cohen's d, Pearson's r), indicating how they were calculated

Our web collection on statistics for biologists contains articles on many of the points above.

Software and code

Policy information about availability of computer code

Data collection	Electrophysiology data were collected using WinWCP (Strathclyde Electrophysiology Software). 2-photon imaging data were acquired using ScanImage (custom-made MATLAB (Mathworks) code).
Data analysis	Slice electrophysiology data were analyzed using Clampfit 10.0 (Molecular Devices) and custom-made MATLAB (Mathworks) code. Images were analyzed using ImageJ software and custom-made MATLAB code.

For manuscripts utilizing custom algorithms or software that are central to the research but not yet described in published literature, software must be made available to editors and reviewers. We strongly encourage code deposition in a community repository (e.g. GitHub). See the Nature Portfolio guidelines for submitting code & software for further information.

Data

Policy information about availability of data

All manuscripts must include a data availability statement. This statement should provide the following information, where applicable:

- Accession codes, unique identifiers, or web links for publicly available datasets
- A description of any restrictions on data availability
- For clinical datasets or third party data, please ensure that the statement adheres to our policy

All source data are provided with this paper. Raw electrophysiology and imaging datasets are available from the corresponding authors upon request. All the code

and analyses used in this study have been deposited at https://doi.org/10.5281/zenodo.7761178. Plasmids for our newly generated viral constructs have been deposited at https://www.addgene.org/plasmids/articles/28225278/.

Human research participants

Policy information about studies involving human research participants and Sex and Gender in Research.

Reporting on sex and gender	N/A
Population characteristics	N/A
Recruitment	N/A
Ethics oversight	N/A

Note that full information on the approval of the study protocol must also be provided in the manuscript.

Field-specific reporting

Please select the one below that is the best fit for your research. If you are not sure, read the appropriate sections before making your selection.

☒ Life sciences ☐ Behavioural & social sciences ☐ Ecological, evolutionary & environmental sciences

For a reference copy of the document with all sections, see nature.com/documents/nr-reporting-summary-flat.pdf

Life sciences study design

All studies must disclose on these points even when the disclosure is negative.

Sample size	Power analysis was used to determine sample sizes, using the formula N = [Z*S / E]^2, where Z is the statistical significance level, S is the standard deviation, and E is the margin of error. For example: in determining N for an LTD experiment, one could used Z = 1.96 (corresponding to p = 0.05), S = 6.7% (realistic std based on previous LTD experiments conducted in the lab), and E = 5% (we want statistical power to determine a 5% difference in LTD magnitude between samples). Thus, N = [1.96 *0.067 / 0.05]^2 = ~7 cells needed.
Data exclusions	Electrophysiology data were excluded only if the recorded cells failed to meet the required criteria of maintenance of access resistance below 25 MΩ and less than 20% change throughout the recording. Animals were excluded based on incorrect targeting of virus.
Replication	All experiments were repeated in a minimum of three cohorts. All attempts at replication were successful.
Randomization	Age- and sex-matched cohorts of mice were allocated into experimental groups and then blinded to the experimenter, such that the order and identity of experimental conditions were not known to the experimenter.
Blinding	Experimenters were blinded to mouse genotypes during experiments.

Reporting for specific materials, systems and methods

We require information from authors about some types of materials, experimental systems and methods used in many studies. Here, indicate whether each material, system or method listed is relevant to your study. If you are not sure if a list item applies to your research, read the appropriate section before selecting a response.

Materials & experimental systems

n/a	Involved in the study	
☐	☒	Antibodies
☐	☒	Eukaryotic cell lines
☒	☐	Palaeontology and archaeology
☐	☒	Animals and other organisms
☒	☐	Clinical data
☒	☐	Dual use research of concern

Methods

n/a	Involved in the study	
☒	☐	ChIP-seq
☒	☐	Flow cytometry
☒	☐	MRI-based neuroimaging

Antibodies

Antibodies used	anti-α-synuclein (1:1000, BD Biosciences, #610786), anti-GFP (1:100, Abcam, ab5450), anti-mouse (1:2000, Thermo Scientific,

Antibodies used	A32728), anti-goat (1:2000, Invitrogen, A-11055), streptavidin (1:1000, Invitrogen, S32355), anti-GFP (1:1000, Santa Cruz Biotechnology, sc-9996), anti-mouse IgG HRP antibody (1:5000, Santa Cruz Biotechnology, SC-516102-CM)
Validation	α-synuclein antibody (BD Biosciences, #610786) was validated by the manufacturer using western blot analysis of rat brain lysate (we also validated using KO mice lacking α-synuclein, which showed no immunoreactivity when assayed with the same secondary antibody). GFP antibody (Abcam, ab5450) was validated by the manufacturer using "knockout edited cell lines for gold-standard validation". anti-GFP (Santa Cruz Biotechnology, sc-9996) was validated by the manufacturer using immunofluorescence staining of methanol-fixed COS cells transfected with GFP fusion protein (or without).

Eukaryotic cell lines

Policy information about cell lines and Sex and Gender in Research

Cell line source(s)	293T cell line
Authentication	Authentication was performed via PCR
Mycoplasma contamination	Cell lines tested negative for mycoplasma contamination
Commonly misidentified lines (See ICLAC register)	N/A

Animals and other research organisms

Policy information about studies involving animals; ARRIVE guidelines recommended for reporting animal research, and Sex and Gender in Research

Laboratory animals	All experiments were performed in accordance with protocols approved by the Stanford University Animal Care and Use Committee in keeping with the National Institutes of Health's Guide for the Care and Use of Laboratory Animals. Animals were kept at a 12hr:12hr light/dark cycle at a room temperature of 22ºC with humidity control (30-70%). Both male and female mice were used for all experiments at ~3-months old (P70-P100), with the exception of recordings from aged mice (16-18 months old). Syn-tKO mice (α-Syn-/-;β-Syn-/-;γ-Syn-/-) were generated as previously described43. WT C57BL/6 mice were maintained as controls, and Syn-tKO mice were back-crossed to C57BL/6 every 6-10 months in order to maintain a consistent background between Syn-tKO and WT lines. α-Syn-KO (α-Syn-/-) and βγ-Syn-KO (β-Syn-/-; γ-Syn-/-) were generated from these backcrosses. Stereotaxic injections were performed 2-6 weeks before recordings.
Wild animals	The study did not involve wild animals.
Reporting on sex	Both male and female mice were used in this study. For all experiments, littermates used were approximately 50/50 - male/female.
Field-collected samples	The study did not involve samples collected from the field.
Ethics oversight	All experiments were performed in accordance with protocols approved by the Stanford University Animal Care and Use Committee in keeping with the National Institutes of Health's Guide for the Care and Use of Laboratory Animals.

Note that full information on the approval of the study protocol must also be provided in the manuscript.
