# Supplementary figures and images for: Postsynaptic synucleins mediate endocannabinoid signaling
Source: Nat Neurosci. 2023 May 29;26(6):997–1007. doi: 10.1038/s41593-023-01345-0 (PMC10244176; doi:10.1038/s41593-023-01345-0)

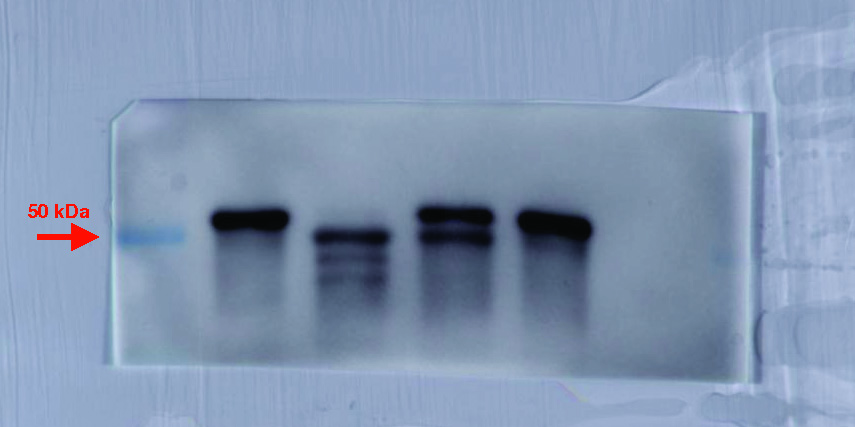

Supplement: Source Data Extended Data Fig. 9 — Unprocessed gel for Extended Data Fig. 9a [file 41593_2023_1345_MOESM18_ESM.jpg]
